# Solvent-free amide bond formation using a variety of methoxysilanes as coupling agent†

**DOI:** 10.1039/d2ob00589a

**Published:** 2022-04-14

**Authors:** Thomas Lainer, Frank Czerny, Michael Haas

**Affiliations:** Institute of Inorganic Chemistry, Graz University of Technology Stremayrgasse 9 8010 Graz Austria michael.haas@tugraz.at https://www.staff.tugraz.at/michael.haas/; Department of Chemistry, Metalorganics and Inorganic Materials, Technische Universität Berlin Strasse des 17. Juni 135 Sekr. C2 10623 Berlin Germany

## Abstract

A solvent-free procedure for the formation of amides without exclusion of air and moisture is described. Using tetramethoxysilane 1, hexamethoxydisilane 2 and dodecamethoxy-neopentasilane 3 as coupling agent carboxylic acids and amines are reacted to form amides in good to excellent yields. The formation of these amides was confirmed by NMR spectroscopy and mass spectrometry. Remarkably, neopentasilane 3 exceeds the performance of the currently used monosilanes as coupling agent in terms of group tolerance and yield.

## Abstract 2

A new solvent-free procedure for the formation of amide bonds without exclusion of air and moisture is described. As coupling agent tetramethoxysilane 1, hexamethoxydisilane 2 and dodecamethoxyneopentasilane 3 were used.

## Graphical abstract

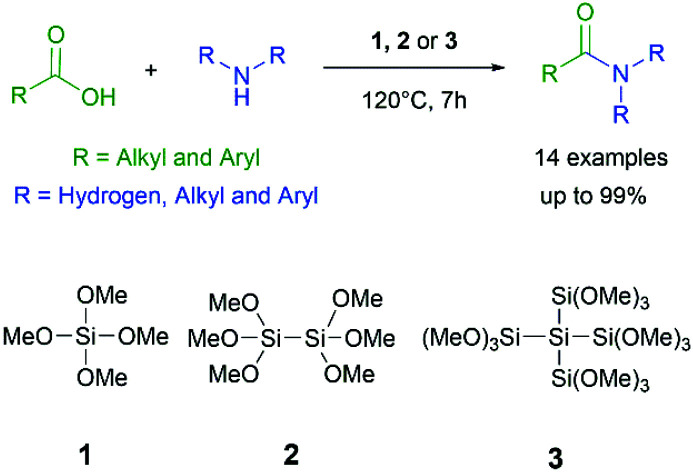

The amide bond formation is one of the most performed transformations in organic chemistry.^1^ The applications range from pharmaceutical products,^2–4^ agrochemicals,^5^ polymers,^6^ hydrogels^7^ and many more. Moreover, this reaction is performed on multiple kilogram scale by the chemical industry. Due to the versatility of this compound class it is not surprising that the number of catalysts and coupling agents is on the steady rise. Traditional methods require pre-activation of the carboxylic acid moiety and the use of stoichiometric coupling agents with additives.^8^ Poor atom economy, high costs as well as toxic and hazardous chemicals^9^ gave rise to copious alternatives over the last decades. The first active silicon reagent was first reported by Chan in 1969.^10^ Liskamp and Mukaiyama continued the research based on reagents derived from SiCl_4_.^11^ Furthermore, Charette *et al.* reported the usage of 9-silafluorenyl dichlorides as efficient reagent.^12^ It was also demonstrated that hydrosilanes like PhSiH_3_,^13^ Ph_2_SiH_2_,^14^ and HSi(OCH(CF_3_)_2_)_3_^15^ are possible reagents for the amide bond formation. In this context, Mukaiyama and Sheppard found that tetramethoxysilane 1 and tetraethoxysilane gave only low conversion when used as stoichiometric reagents in THF^15^ and acetonitrile.^16^ Braddock, Lickiss *et al.* used toluene instead of polar solvents and obtained high yields of pure amide products after the work up procedure without the necessity of a chromatographic purification. A silyl ester intermediate could be detected by *in situ* NMR-spectroscopy (Scheme 1).^17^

**Scheme 1:**
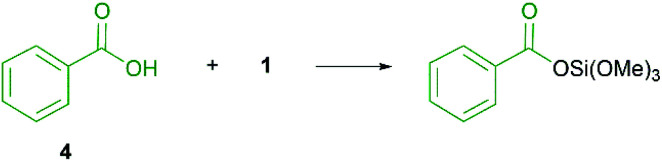
Postulated silyl ester formation as intermediate.

Due to these numerous successful examples of silicon coupling agents and our expertise in polysilane chemistry we decided to investigate the peptide bond formation with the polysilanes hexamethoxydisilane 2 and dodecamethoxyneopentasilane 3. The reason for the investigation 2 and 3 was the presence of Si–Si bonds in the molecules, which are known to have a lower bond energy than Si–O bonds. In the scope of our research we selected the previously reported tetramethoxysilane 1 as a benchmark compound (Chart 1).

**Chart 1:**
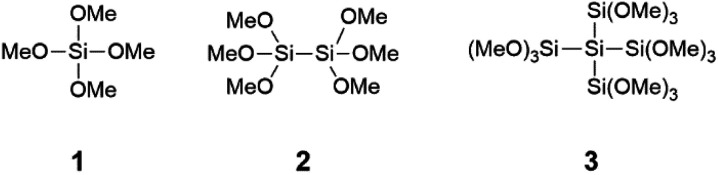
Used methoxysilanes as coupling agents.

We used benzoic acid as our standard acid and benzylamine as standard amine to determine the optimal conditions (Scheme 2). To keep the experiment as simple as possible the reaction vessel was a GC-vial with a needle as pressure and methanol release.

**Scheme 2:**
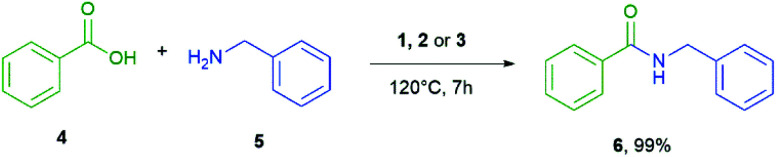
Amide bond formation of the optimization reaction.

In Table 1 the optimization of the reaction conditions are depicted. Compound 3 was utilized to find the correct time and temperature conditions to obtain an ideal outcome of the reaction. As shown in Table 1 the reaction of the acid and amine also proceeds at 80 °C with coupling agent 3, but in very low yields. However, at 120 °C and 7 hours 99% yield of the amide 6 could be achieved (20% loading of 3). To determine the difference of the used alkoxysilanes, the time and temperature were set for all three compounds. As expected, the loading of the silanes in the reaction increased with decreasing number of trimethoxysilyl groups in the compound. Compound 2 showed the same performance as 3 with 60% loading. For compound 1 120 mol% were needed at the determined time and temperature to obtain the optimum yield.

**Table 1:** Optimization of the reaction conditions

Coupling agent	Loading [mol%]	Temperature [°C]	Yield [%]
3	10%	80	20
3	10%	120	81
3	15%	120	90
3	20%	80	30
3^a^	20%	120	70
3	20%	120	99
2	40%	120	93
2	50%	120	95
2	60%	120	99
1	40%	120	70
1	80%	120	81
1	120%	120	99

a Reaction time = 3 h.

Once the conditions were set, the next step was to vary the carboxylic acid. In order to obtain information about the group tolerance we did not alter the reaction conditions. As shown in Scheme 3 and Table 2 this synthetic protocol can also be applied to aliphatic carboxylic acids, secondary carboxylic acids and substituted benzoic acids. The amide 7 could be obtained in high yields with all three coupling agents. The yield of 7 with the aliphatic backbone is almost comparable with our optimized standard reaction. In contrast for the amides 8–13 coupling efficiency strongly depends on the used silane. In general, the yields with use of the monosilane 1 and the disilane 2 as coupling agents are comparable and in the same range, but significantly lower than for neopentasilane 3. Moreover, the coupling agent 3 showed a higher group tolerance and higher yields in all cases.

**Scheme 3:**
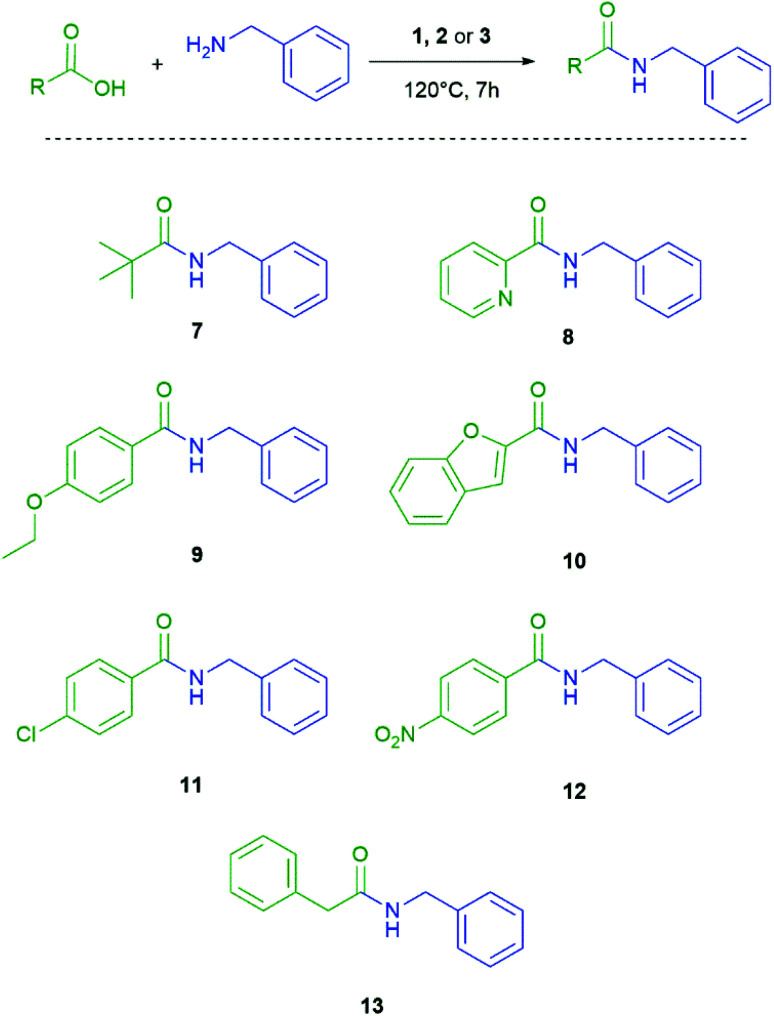
Different carboxylic acids coupled with benzylamine.

**Table 2:** Yield comparison with different carboxylic acids

Amide	Coupling agent		
	1^a^	2^b^	3^c^
	Yield		
7	95%	86%	95%
8	66%	74%	94%
9	74%	71%	74%
10	51%	61%	81%
11	81%	54%	82%
12	66%	57%	83%
13	95%	95%	98%

a Coupling agent loading 120%.

b Coupling agent loading 60%.

c Coupling agent loading 20%.

Continuing with our investigation we also altered the amines for the amide bond formation (Scheme 4 and Table 3). The formation of amides by the coupling of primary and secondary amines with benzoic acid was observed with all investigated coupling agents. In line with our previous observations the neopentasilane 3 as coupling agent provides the highest yield from 82% to 93%. The silanes 1 and 2 are again in the same range except for the secondary amine 16 where 2 shows a similar yield as 3. For compound 16 the conditions had to be altered because of the low boiling point of piperidine. In this case the GC-vial had no pressure release. We also tried to obtain an imide 14 by coupling benzamide and benzoic acid, but no silane could catalyse the product formation with these conditions.

**Scheme 4:**
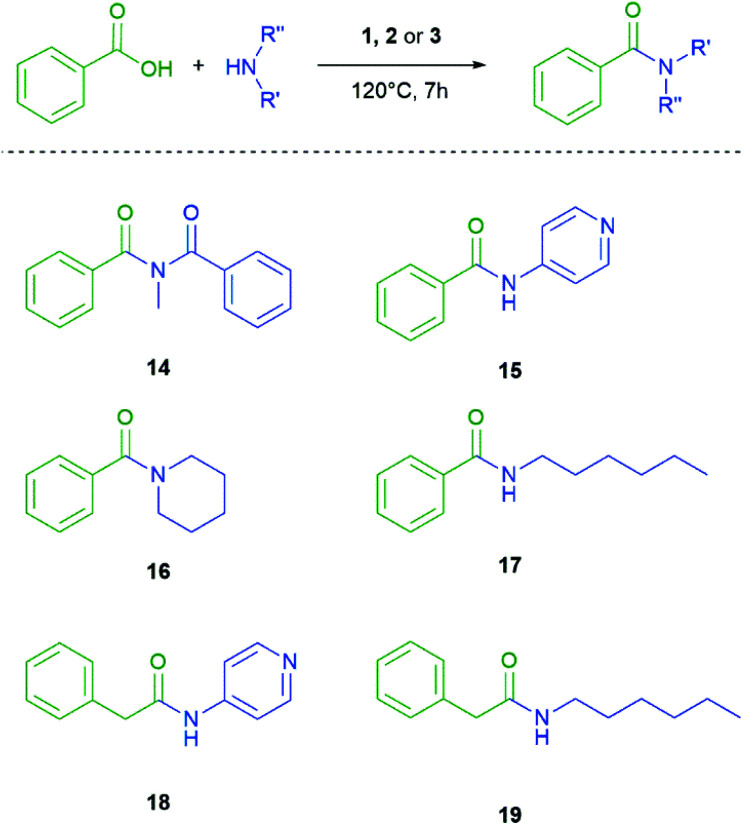
Different amines coupled with benzoic acid.

**Table 3:** Yield comparison with different amides

Amide	Coupling agent		
	1^a^	2^b^	3^c^
	Yield		
14	0%	0%	0%
15	63%	63%	82%
16	65%	90%	93%
17	70%	60%	85%
18	62%	60%	83%
19	68%	61%	82%

a Coupling agent loading 120%.

b Coupling agent loading 60%.

c Coupling agent loading 20%.

On the basis of the presented data above we could determine the equivalents needed for the best performance of our coupling agents. In the case of the monosilane 1 1.2 equivalents and in the case of 2 0.6 equivalents were needed, which means that only one methoxy group of each silicon atom reacts with the acid to form the ester. For the neopentasilane 3 0.2 equivalents were needed for the best performance, which consequently means there are five methoxy groups involved. However, this would be against the common trend, that only one methoxy group on each silicon atom reacts with the acid. The more plausible explanation is that there is a second reaction pathway involved. As Höfler and Jannach already discovered in 1975 methanol is able to cleave the silicon–silicon bond of branched polymethoxysilanes (Scheme 5).^18^ Taking this into account, the quaternary silicon-atom acts as hydrosilane which can also act as coupling agent for the amide bond formation.^14^ Consequently, neopentasilane 3 has five reactive sides (four trimethoxy groups and one hydride), which can act as coupling agents.

**Scheme 5:**
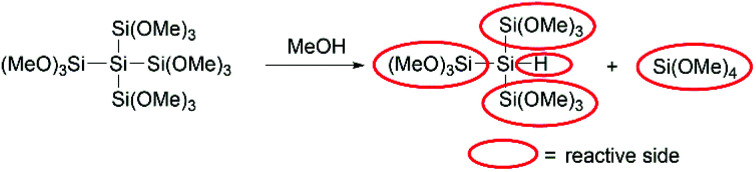
Plausible mechanism of the fragmentation of 3.

To obtain a structural information about the formed polysiloxane after the coupling reaction, we reacted benzoic acid and benzylamine with 20 mol% of 3 and isolated the insoluble polysiloxane by washing the reaction mixture with hot toluene and THF to remove the formed amide (Scheme 6).

**Scheme 6:**
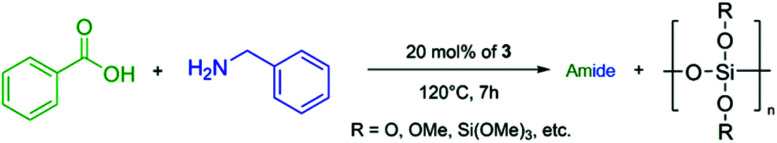
Isolation of the insoluble polysiloxane.

To analyse this polysiloxane, IR spectroscopy was performed. The IR spectrum shows a strong band centered at 1057 cm^−1^, which can be attributed to *ν*_SiO_ vibrations. Another group of bands can be found between 2800–3100 cm^−1^, which can be assigned to *ν*_CH_ vibrations, indicating the presence of methoxy groups in the material. Consistently, elemental analysis of the polysiloxane reveals 24 wt% of C and 5 wt% of H. SEM mapping showed a homogeneous distribution of each element (see ESI†). Furthermore, the polysiloxane was analysed by X-ray photoelectron spectroscopy (XPS). The Si 2p spectrum shows a peak at 103.4 eV for Si 2p_3/2_, which is in the typical range of Si(iv) compounds.^19^ Since 3 is a mixed valence compound with silicon in the oxidation states of 0 and 3, XPS analysis reveals an oxidation process during the reaction. ^1^H and ^13^C solid-state NMR spectroscopy indicates, in good agreement with the aforementioned methods, the presence of methoxy groups in the material. ^29^Si CPMAS NMR spectroscopy shows peaks at −86.37 ppm, −94.43 ppm, −102.87 ppm and −111.35 ppm, which can be assigned to Q_1_, Q_2_, Q_3_ and Q_4_ sites, respectively.^20,21^ Q_*n*_ (*n* = 0, 1, 2, 3, 4) represents a Si atom bonded through oxygen to *n* other Si atoms, therefore describing the degree of crosslinking within the network.^21^ Additionally, no signals from the starting material (−35.9 ppm Si(OMe)_3_ and −172.2 ppm Si*q*) could be detected, which indicate that all Si–Si bonds were broken. Taking all gathered analytic data into account, we propose the structure shown in Chart 2 for the polysiloxane.

**Chart 2:**
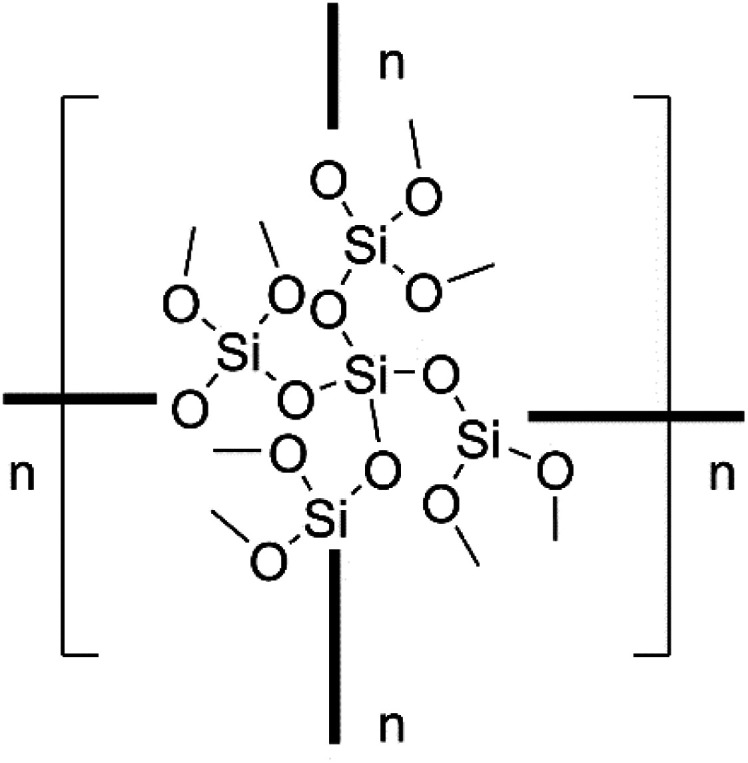
Proposed structure of the polysiloxane.

Large scale production is also crucial for amide synthesis, so we performed a multigram synthesis with 1.00 g benzoic acid and 0.90 mL benzylamine and 20 mol% of compound 3 using standard conditions. The obtained 1.69 g of 6 (98% yield) can be feasibly compared to the small gram synthesis. In order to exclude that the presence of air and moisture has any influence on the outcome of the reaction, we also performed the reactions in a glove box and observed no change in the reactivity and yield of the desired products.

In conclusion we reported on a solvent-free procedure for the formation of different kinds of amides. As coupling agents, we used three different kinds of methoxysilanes. This one-pot synthesis afforded the amides in good to excellent yield without the exclusion of air and moisture. The present work also demonstrated the possibility for the amide synthesis in multigram scale. Moreover, we could determine the equivalents needed for the best performance of our coupling agents. In order to obtain a structural information, the formed polysiloxane was isolated and analysed with a variety of spectroscopic methods including scanning electron microscopy (SEM) and X-ray photoelectron spectroscopy (XPS).

## Conflicts of interest

The authors declare no conflict of interest.

## Supplementary Material

OB-020-D2OB00589A-s001
