# Supplementary material for: Solvent-free amide bond formation using a variety of methoxysilanes as coupling agent
Source: Org Biomol Chem. 2022 Apr 14;20(18):3717–20. doi: 10.1039/d2ob00589a (PMC9092949; doi:10.1039/d2ob00589a)
Supplement: OB-020-D2OB00589A-s001 [file OB-020-D2OB00589A-s001.pdf]

Electronic Supplementary Material (ESI) for Organic & Biomolecular Chemistry.
This journal is © The Royal Society of Chemistry 2022

## Supporting Information

# Solvent-Free Amide Bond Formation Using a Variety of Methoxysilanes as Coupling Agent

Thomas Lainer,[a] Frank Czerny[b] and Michael Haas*[a]

[a] Institute of Inorganic Chemistry, Graz University of Technology, Stremayrgasse 9, A-8010 Graz, Austria

[b] Department of Chemistry: Metalorganics and Inorganic Materials, Technische Universität Berlin, Strasse des 17. Juni 135, Sekr.C2, 10623 Berlin, Germany

# Table of Content

Experimental Procedures ........ 3

General Considerations ........ 3

General Experimental Procedure ........ 4

Analytic Section ........ 4

Table 1 Spectroscopic Characterization of Compound (6-19) ........ 4

NMR and GC-MS-spectra ........ 7

Figure S1 $^1$H-NMR of **6** ........ 7

Figure S2 $^{13}$C-NMR of **6** ........ 8

Figure S3 GC-MS of **6** ........ 9

Figure S4 GC-MS of **7** ........ 10

Figure S5 $^1$H-NMR of **8** ........ 11

Figure S6 $^{13}$C-NMR of **8** ........ 12

Figure S7 GC-MS of **8** ........ 13

Figure S8 $^1$H-NMR of **9** ........ 14

Figure S9 $^{13}$C-NMR of **9** ........ 15

Figure S10 GC-MS of **9** ........ 16

Figure S11 $^1$H-NMR of **10** ........ 17

Figure S12 $^{13}$C-NMR of **10** ........ 18

Figure S13 $^{13}$C-NMR of **10** ........ 19

Figure S14 $^1$H NMR of **11** ........ 20

Figure S15 $^{13}$C-NMR of **11** ........ 21

Figure S16 GC-MS of **11** ........ 22

Figure S17 $^1$H NMR of **12** ........ 23

Figure S18 $^{13}$C-NMR of **12** ........ 24

Figure S19 GC-MS of **12** ........ 25

Figure S20 $^1$H-NMR of **13** ........ 26

Figure S21 $^{13}$C-NMR of **13** ........ 27

Figure S22 GC-MS of **13** ........ 28

Figure S23 $^1$H NMR of **15** ........ 29

Figure S24 $^{13}$C-NMR of **15** ........ 30

Figure S25 GC-MS of **15** ........ 31

Figure S26 $^1$H NMR of **16** ........ 32

Figure S27 $^{13}$C-NMR of **16** ........ 33

Figure S28 GC-MS of **16** ........ 34

Figure S29 $^1$H NMR of **17** ........ 35

Figure S30 $^{13}$C-NMR of **17** .......... 36
Figure S31 GC-MS of **17** .......... 37
Figure S32 $^{1}$H-NMR of **18** .......... 38
Figure S33 $^{13}$C-NMR of **18** .......... 39
Figure S34 GC-MS of **18** .......... 40
Figure S35 $^{1}$H-NMR of **19** .......... 41
Figure S36 $^{13}$C-NMR of **19** .......... 42
Figure S37 GC-MS of **19** .......... 43
Figure S38 SEM/EDX mapping of the polysiloxane on a Si wafer .......... 44
Figure S39 High-resolution XPS Si2p spectrum of the .......... 44
Figure S40 Solid state $^{1}$H NMR spectrum of the .......... 45
Figure S41 $^{13}$C CPMAS NMR spectrum of the polysiloxane .......... 45
Figure S42 $^{29}$Si CPMAS NMR spectrum of the polysiloxane .......... 46

# Experimental Procedures

## General Considerations

Commercial reagents were used as purchased unless otherwise noted. $^{1}$H (299.95 MHz) and $^{13}$C (75.43 MHz) NMR spectra were either recorded on a Varian INOVA MHz 300 or a Varian Mercury MHz 300 spectrometer in $CDCl_3$ or DMSO-$d_6$ solution and referenced versus TMS using the internal $^{2}$H-lock signal of the solvent. Solid state NMR measurements were carried out on a Bruker Avance 400 MHz. Chemical shifts (δ) are given in ppm.

Scanning electron microscopy (SEM) was performed on a GeminiSEM500 NanoVP microscope (ZEISS) integrated with an EDX detector (Bruker Quantax XFlash® 6/60). Data handling and analysis were achieved with the software package EDAX. The SEM experiments were conducted at the Zentrum für Elektronenmikroskopie (ZELMI) of the TU Berlin.

X-ray photoelectron spectroscopy (XPS) was measured on K-Alpha™ + X-ray Photoelectron Spectrometer System (Thermo Scientific) with Hemispheric 180 ° dual-focus analyzer with 128-channel detector. X-ray monochromator is micro focused Al-Kα radiation. For the measurement, the powder samples were mounted on conductive carbon tapes in the glovebox and the sample holder was then transferred under vacuum into the spectrometer. The data were collected with an x-ray spot size of 400 µm, 20 scans for survey and 50 scans for regions. Survey spectra were run in the binding energy range of 0-1000 eV. High-resolution spectra of Si2p and O1s were collected.

## General Experimental Procedure

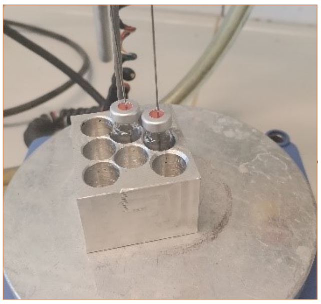

Carboxylic acid (1,64 mmol), amine (1.64) and either coupling agent **1** (120 mol% tetramethoxysilane), **2** (60% hexamethoxydisilane) or **3** (20%

dodecamethoxyneopentasilane) were charged in a gc-vial with a needle as pressure release (picture 1). The heating block was heated for 7 hours at 120°C. Once completed aqueous work up with $K_2CO_3$ took place. The organic solvents were dried over sodium sulphate, filtered and evaporated. The pure compounds could be obtained after column chromatography with heptane/EE 1:1.

**Picture 1**: Experimental Setup

## Analytic Section

Table 1 Spectroscopic Characterization of Compound (6-19)

| | Structure/Yield[(a)] | Spectral Data | Ref. |
|---|---|---|---|
| 6 | 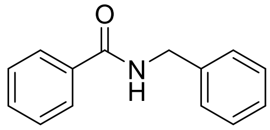 Yield: (1) 99% (2) 99% (3) 99% | $^{1}$H-NMR (CDCl$_3$, 300 MHz): δ 7.73-7.70 (m, 2H, Ar-H), 7.18-7.42 (m, 8H, Ar-H) 6.57 (br s, 1H, N-H) 4.56 (d, 2H); $^{13}$C-NMR (CDCl$_3$, 76 MHz): δ 167.42, 138.20, 134.41, 131.58, 128.82, 128.62, 127.96, 127.66, 126.99, 44.19 MS(70ev): m/z 211.1 | 1 |
| 7 | 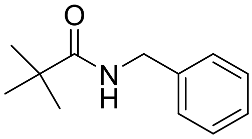 Yield: (1) 95% (2) 86% (3) 95% | $^{1}$H-NMR (CDCl$_3$, 300 MHz): δ 7.2-7.40 (m, 5H, Ar-H), 5.95 (br s, 1H, N-H) 4.45 (d, 2H); 1.2 (s, 9H) $^{13}$C-NMR (CDCl$_3$, 76 MHz): δ 178.20, 138.22, 128.5, 127.58, 43.44, 27.55 MS(70ev): m/z 191.1 | 2 |
| 8 | 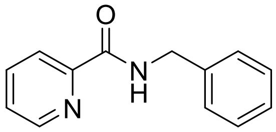 Yield: (1) 66% (2) 74% (3) 94% | $^{1}$H-NMR (CDCl$_3$, 300 MHz): δ 8.45(d, 1H, Ar-H), 8.30 (s, 1H, Ar-H) 8.15 (d, 1H, Ar-H), 7.81 (t, 1H, Ar-H), 7.21 (m, 5H, Ar-H); 4.59 (d, 2H)$^{13}$C-NMR (CDCl$_3$, 76 MHz):δ 164.26, 149.89, 148.11, 138.25, 137.38, 128.73, 127.88, 127.38, 126.15, 122.38, 43.36.MS(70ev): m/z 212.0 | 3 |
| 9 | 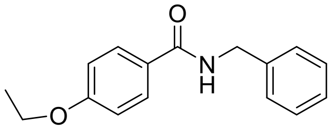 Yield: (1) 74% (2) 71% (3) 74% | $^{1}$H-NMR (CDCl$_3$, 300 MHz): δ 7.64 (d, 2H, Ar-H), 7.22 (m, 5H, Ar-H) 6.91 (d, 2H, Ar-H), 6.02 (s, 1H, N-H), 4.56 (q, 2H, Ar-H), 4.56 (q, 2H, Ar-H), 1.45 (t, 3H, Ar-H); $^{13}$C-NMR (CDCl$_3$, 76 MHz):δ 166.95, 161.51, 138,44, 138.15, 128.78, 128.74, 127.90, 114.22, 63.63, 44.07, 14.15 MS(70ev): m/z 255.1 | 4 |
| 10 | 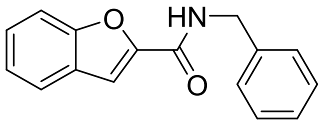 Yield: (1) 51% (2) 61% (3) 81% | $^{1}$H-NMR (CDCl$_3$, 300 MHz): δ 7.57 (d, 1H, Ar-H) 7.14-7.42 (m, 9H, Ar-H), 6.94 (br s, 1H, N-H) 4.56 (d, 2H); $^{13}$C-NMR (CDCl$_3$, 76 MHz):δ 158.85, 154.78, 148.84, 137.84, 128.85, 128.0, 127.76, 126.94, 123.75, 111.75, 110.68, 43.43 MS(70ev): m/z 251.1 | 5 |
| 11 | 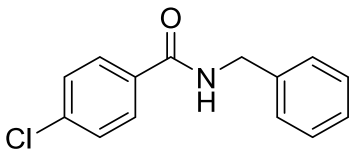 Yield: (1) 66% (2) 54% (3) 82% | $^{1}$H-NMR (CDCl$_3$, 300 MHz): δ 7.19-7.65 (m, 9H, Ar-H), 6.36 (br s, 1H, N-H) 4.55 (d, 2H); $^{13}$C-NMR (CDCl$_3$, 76 MHz):δ 166.11, 137.95, 137.84, 132.76, 128.88, 128.43, 127.98, 127.77, 44.11 MS(70ev): m/z 245.0 | 6 |
| 12 | 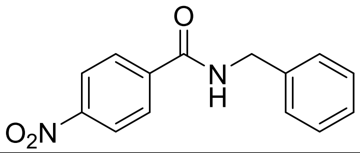 | $^{1}$H-NMR (CDCl$_3$, 300 MHz): δ 8.17 (d, 2H, Ar-H), 7.86 (d, 2H, Ar-H)7.24-7.38 (m, 5H, Ar-H) 6.61 (br s, 1H, N-H) 4.59 (d, 2H); $^{13}$C NMR (76 MHz, CDCl3) δ 170.90, | 7 |

| | | | |
|---|---|---|---|
| | Yield: (1) 66 % (2) 57% (3) 83% | 138.19, 134.85, 129.47, 129.07, 128.67, 127.51, 127.44, 127.41, 43,84, 43,60; MS(70ev): m/z 225.0 | |
| 13 | 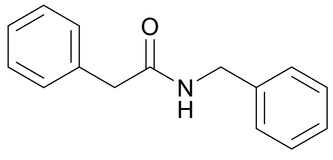  Yield: (1) 95% (2) 95% (3) 98% | $^{1}$H-NMR ($CDCl_3$, 300 MHz): δ 7.08-7.29 (m, 10H, Ar-H), 5.73 (br s, 1H, N-H) 4.31 (d, 2H), 3.53 (s, 2H); $^{13}$C-NMR ($CDCl_3$, 76 MHz): δ 167.42, 138.20, 134.41, 131.58, 128.82, 128.62, 127.96, 127.66, 126.99, 44.19 MS(70ev): m/z 211.1 | 8 |
| 14 | 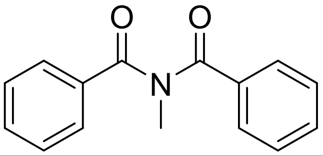  | No reaction | |
| 15 | 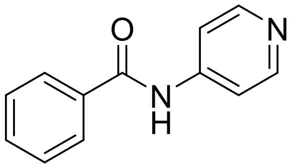  Yield: (1) 63% (2) 63% (3) 82% | $^{1}$H-NMR (DMSO-$d_6$, 300 MHz): δ 10.6 (br s, 1H, N-H), 8.48 (br,s , 2H, Ar-H) 7.97 (m, 2H, Ar-H), 7.87 (m, 2H, Ar-H) 7.58 7.97 (m, 5H, Ar-H); $^{13}$C NMR (76 MHz, DMSO-$d_6$) δ 166.48, 150.29, 145.91, 134.24, 132.10, 128.36, 127.72, 113.70, MS(70ev): m/z 198.1 | 9 |
| 16 | 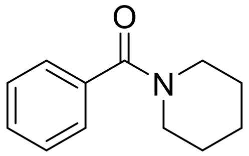  Yield: (1) 65% (2) 90% (3) 93% | $^{1}$H-NMR (DMSO-$d_6$, 300 MHz): δ 7.42 (m, 5H, Ar-H), 3.2-3.5 (m, 4H, Ar-H) 1.59 (m, 6H); $^{13}$C NMR (76 MHz, DMSO-$d_{6,}$) δ 168.83, 136.55, 129.16, 128.33, 126.54, 47.78, 42.45, 25.50, 24.02; MS(70ev): m/z 188.10 | 10 |
| 17 | 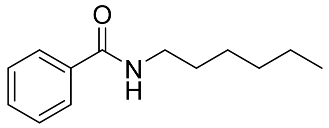  Yield: (1) 70% (2) 60% (3) 85% | $^{1}$H-NMR ($CDCl_3$, 300 MHz): δ 7.34-7.68 (m, 5H, Ar-H),6.18 (bs 1H, N-H) 3.36 (m, 2H, ), 1.53 (m, 2H); 1.25 (m, 6H,) 0.82 (m, 3H)$^{13}$C NMR $^{13}$C NMR (76 MHz, CDCl3) δ 167.23, 134.94, 131.33, 128.58, 126.89, 40.22, 31.80, 29.13, 26.41, 22.64, 13.77.; MS(70ev): m/z 205.10 | 11 |
| 18 | 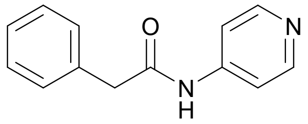  Yield: (1) 62% (2) 60% (3) 83% | $^{1}$H-NMR (DMSO-$d_6$, 300 MHz): δ 10.80 (s, 1H) 8.45 (d, 2H) 7.64 (d, 2H) 7.30 (m, 4H,), 3.73 (s, 2H,) $^{13}$C NMR (76 MHz, DMSO-$d_{6,}$) δ 170.56, 149.15, 146.79, 135.20, 129.21, 128.34, 126.69, 113.29, 43.29,; MS(70ev): m/z 212.0 | 12 |
| 19 | 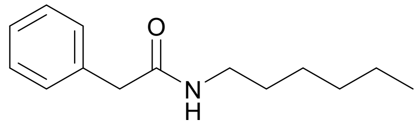  Yield: (1) 68% (2) 61% (3) 82% | $^{1}$H-NMR ($CDCl_3$, 300 MHz): δ 7.17-7.38 (m, 5H, Ar-H),5.38 (bs 1H, N-H), 3.49 (s, 2H, ), 3.11 (m, 2H); 1.33 (m, 2H), 1.15 (m, 6H), 0.78 (m, 3) $^{13}$C NMR $^{13}$C NMR (76 MHz, CDCl3) δ 170.86, 135.14, 129.44, 129.00, 127.30, 43.92, 39.69, 31.38, 29.41, 26.43, 22.50; 13.95 MS(70ev): m/z 219.0 | 13 |

(a) yield with different coupling agent used: (1) tetramethoxysilane (2) hexamethoxydisilane (3) dodecamethoxyneopentasilane

**NMR and GC-MS-spectra**

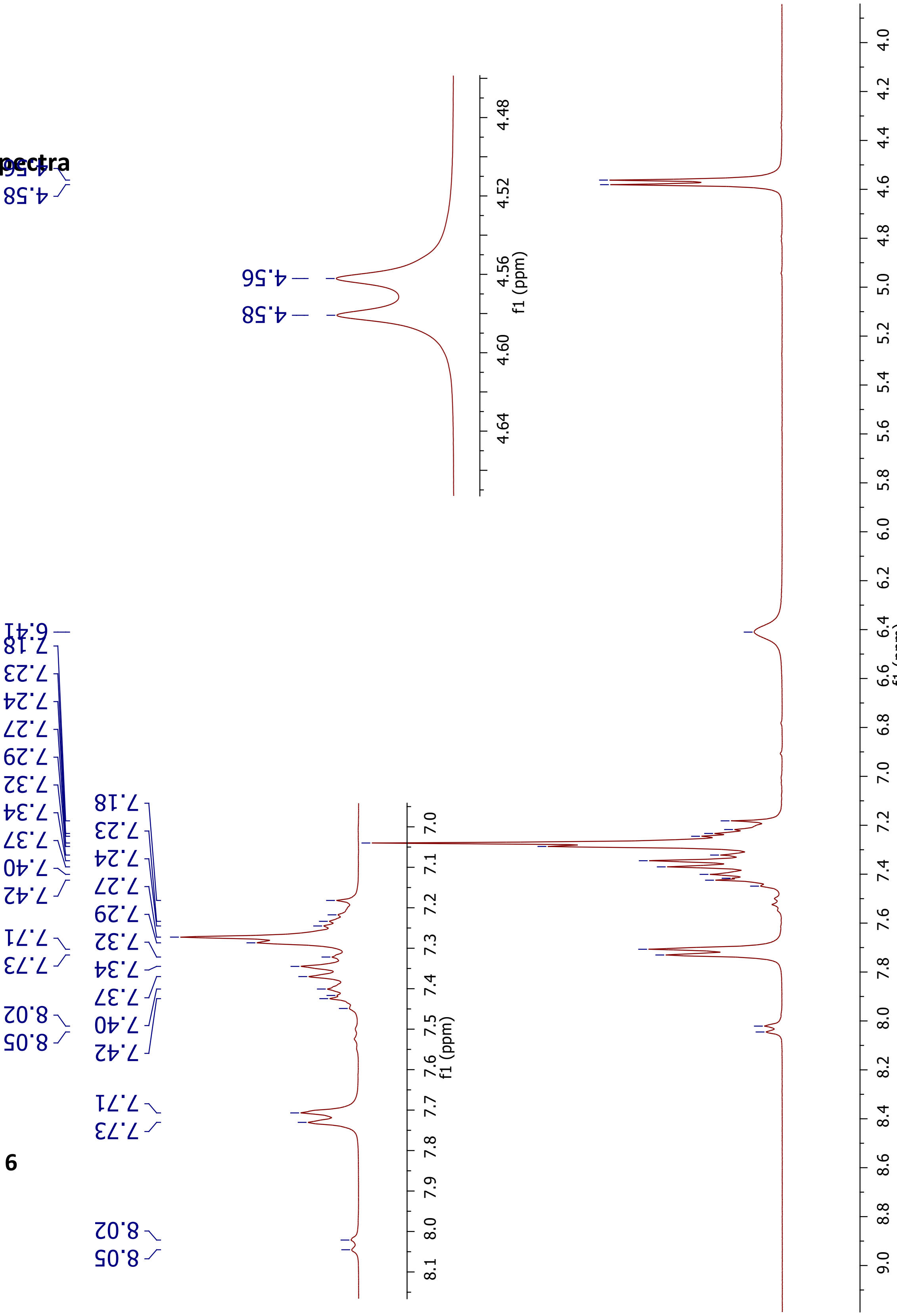


Figure S1 ¹H-NMR of **6**

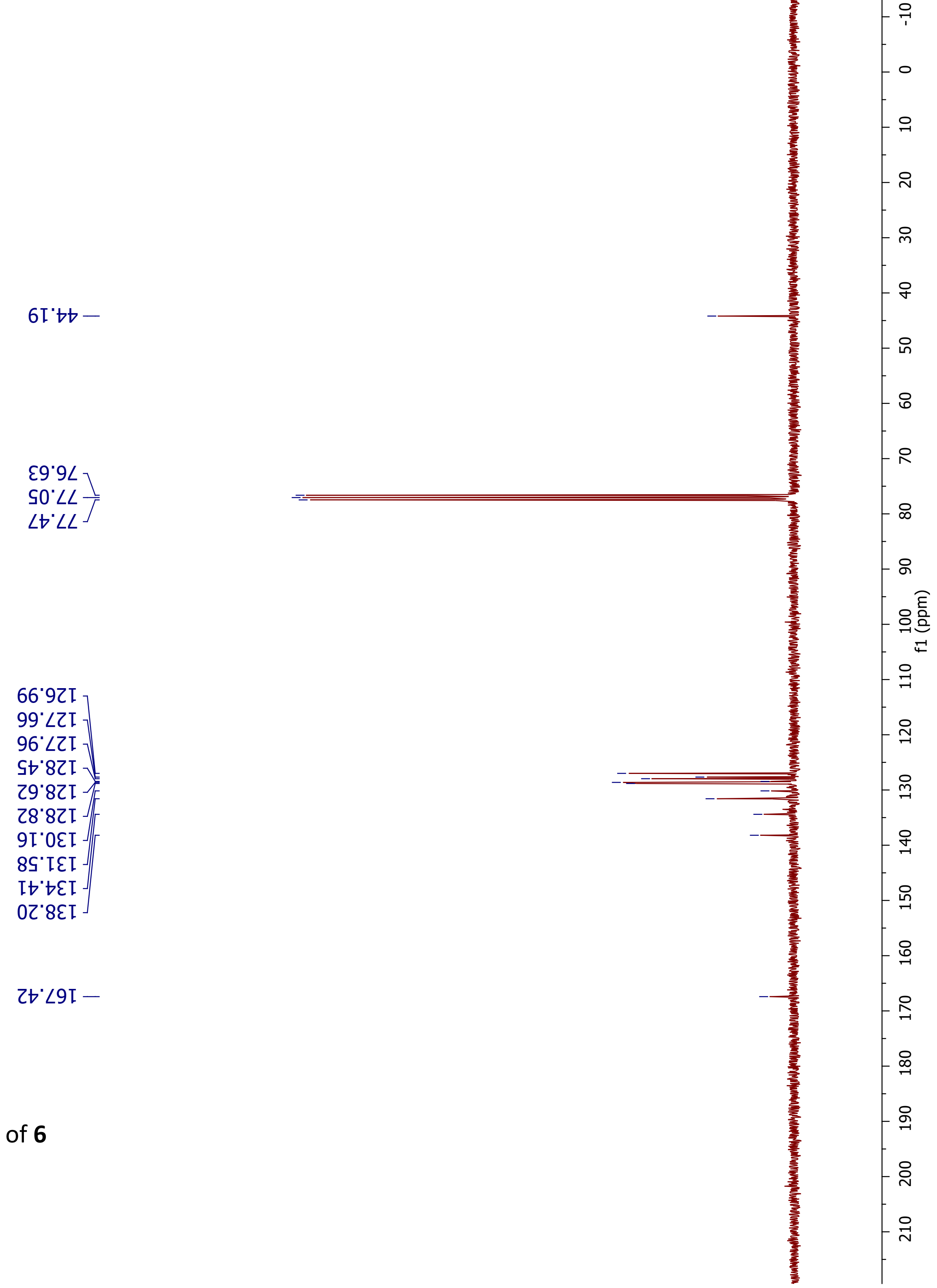


Figure S2 $^{13}$C-NMR of **6**

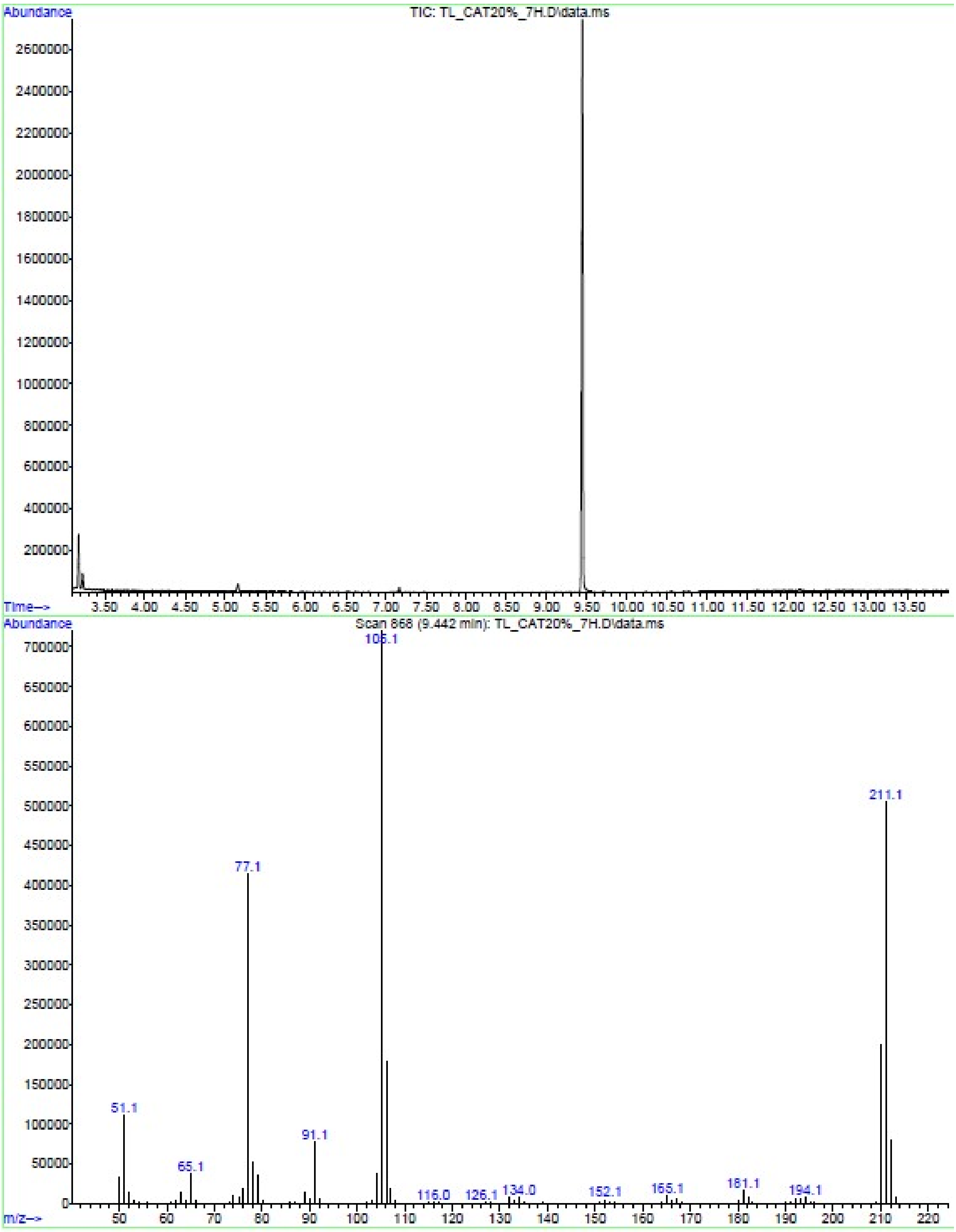


Figure S3 GC-MS of **6**

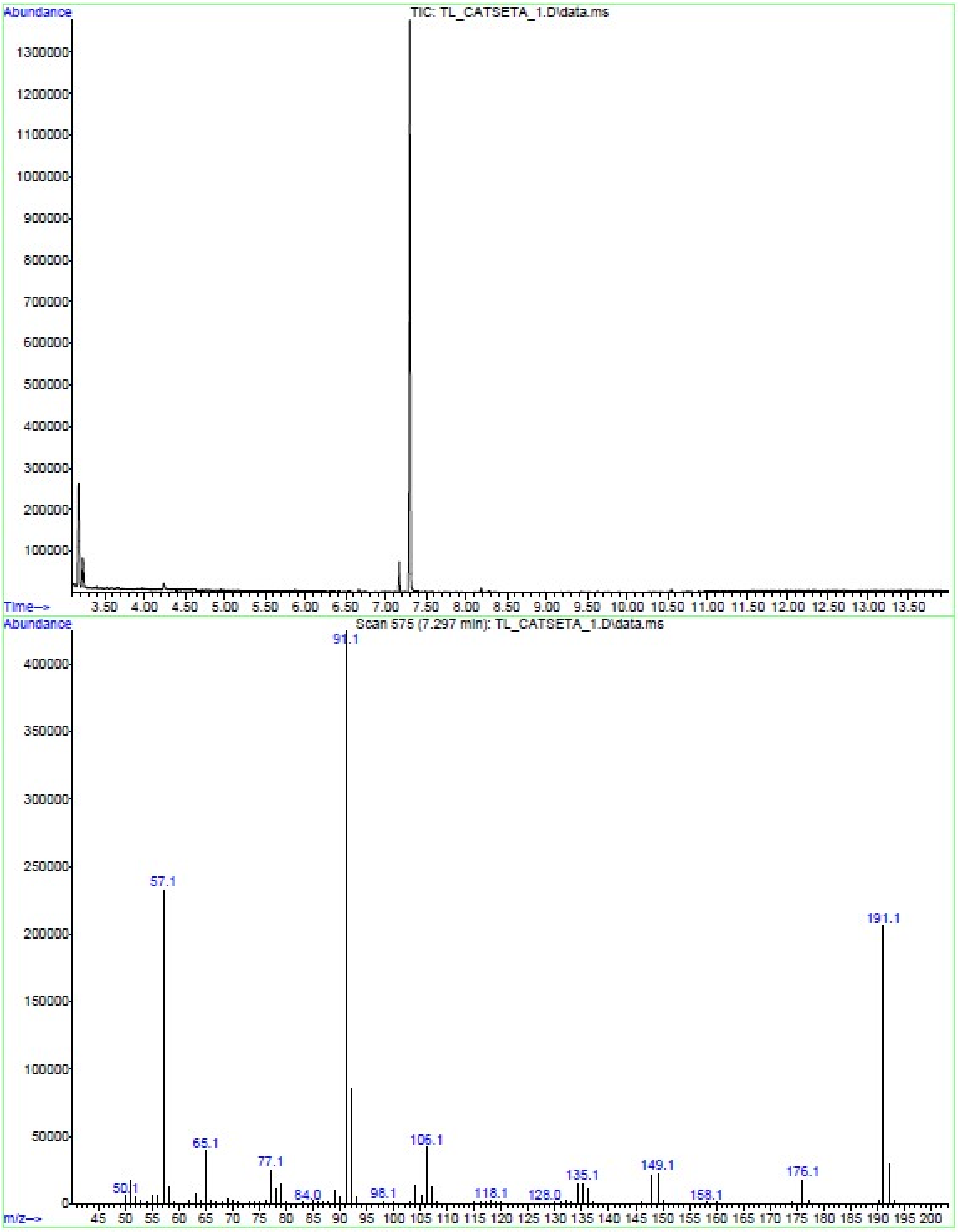


Figure S4 GC-MS of **7**

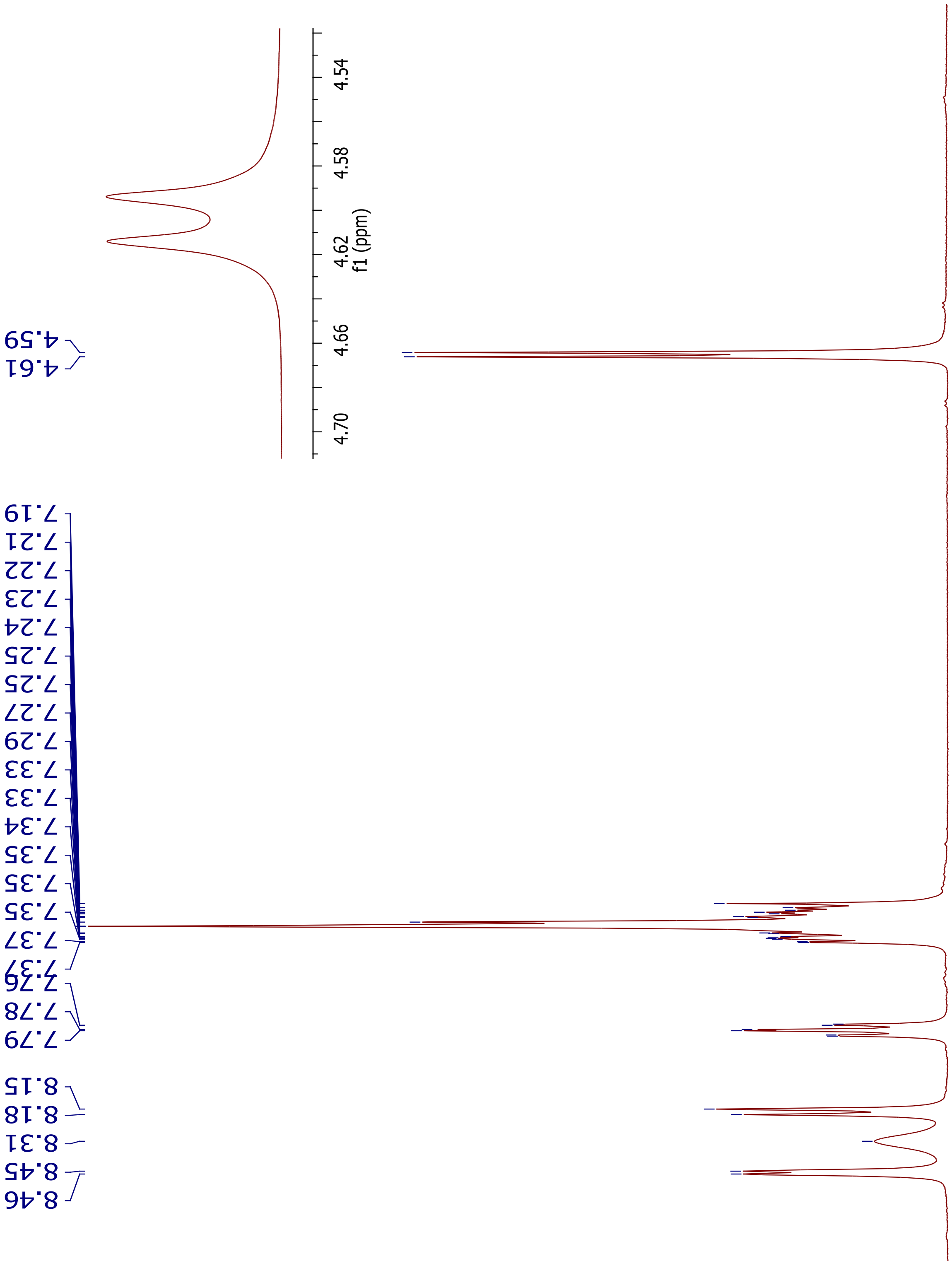


Figure S5 $^{1}$H-NMR of **8**

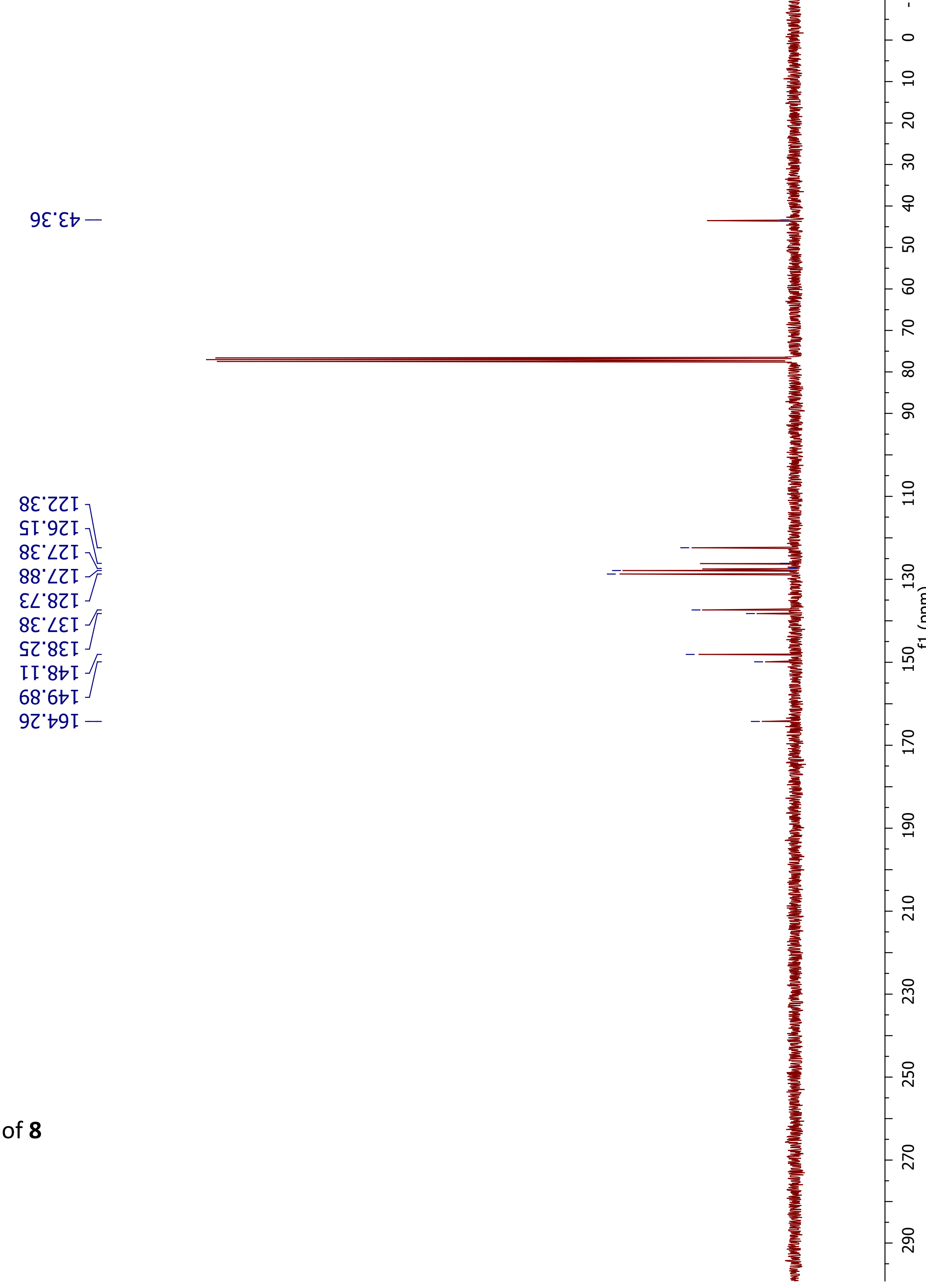


Figure S6 $^{13}$C-NMR of **8**

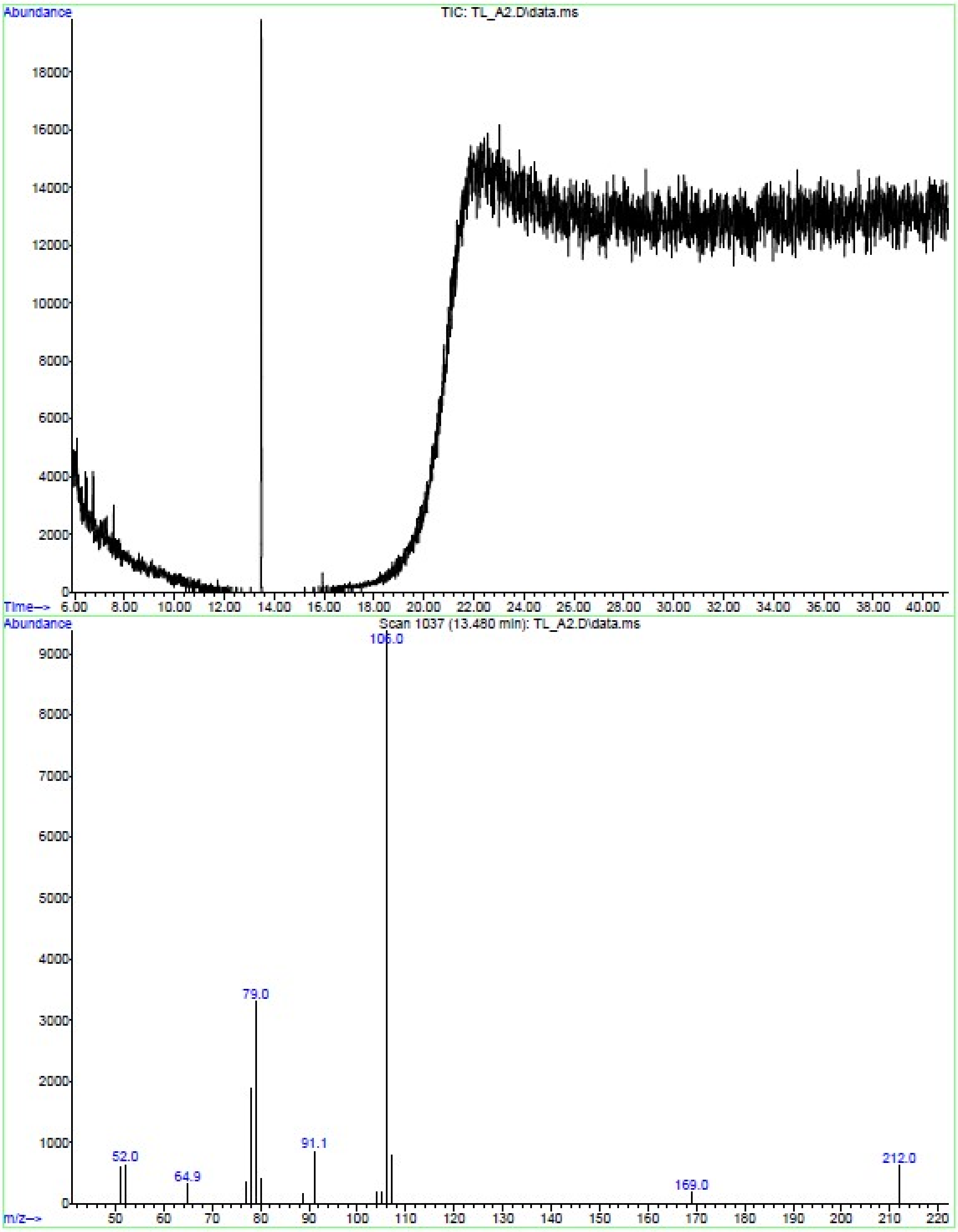


Figure S7 GC-MS of **8**

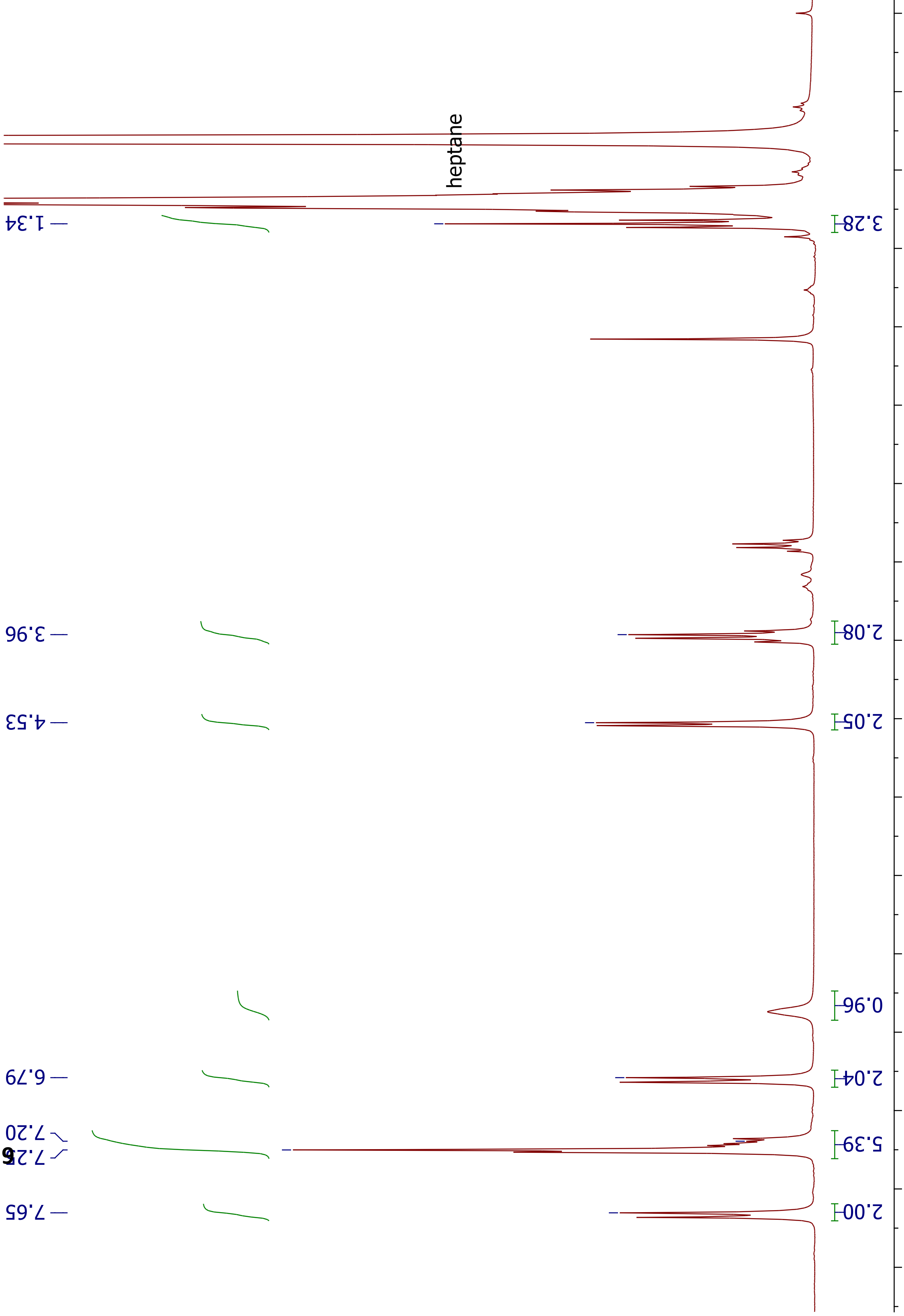


Figure S8 $^{1}$H-NMR of 5

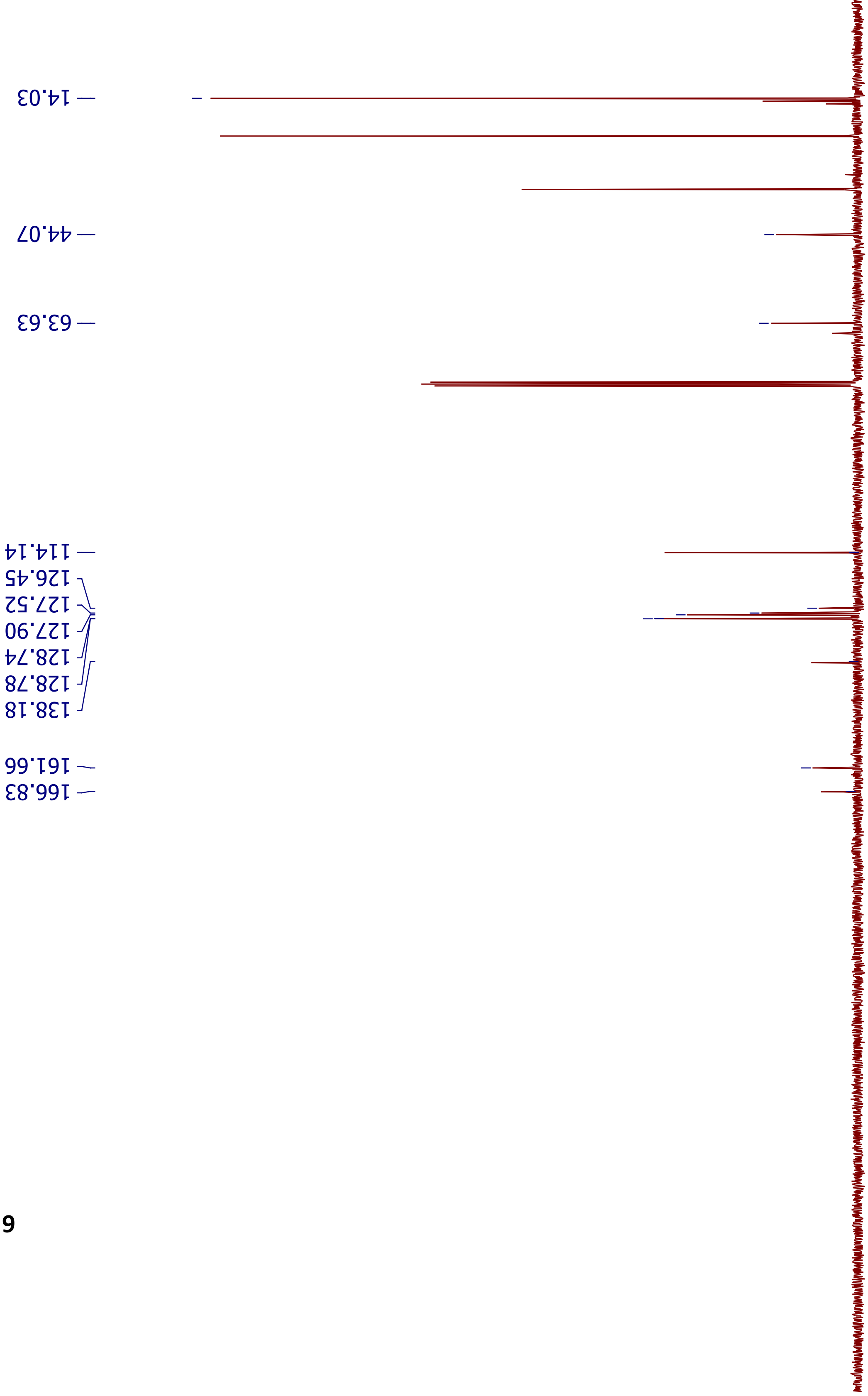


Figure S9 $^{13}$C-NMR of **9**

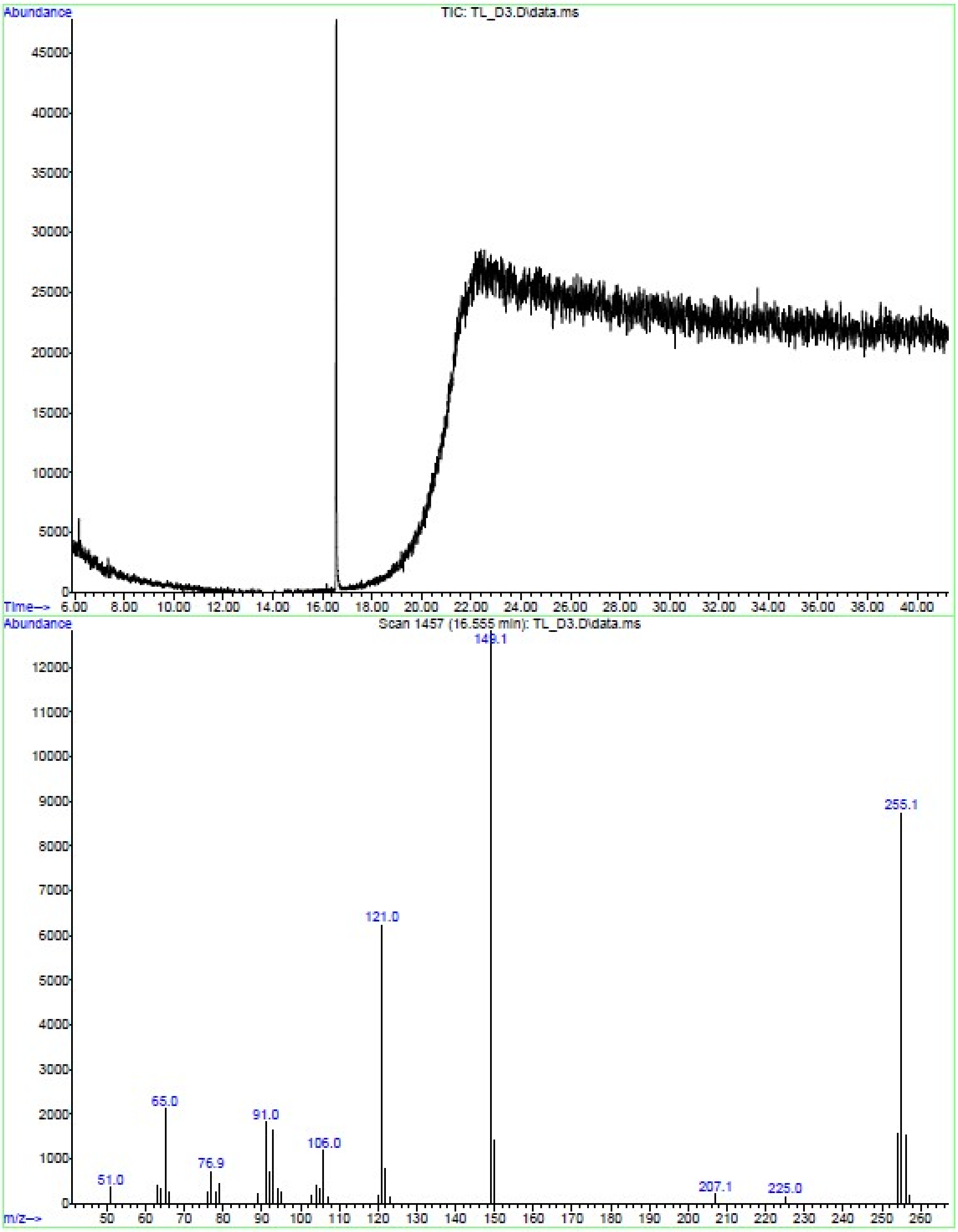


Figure S10 GC-MS of **9**

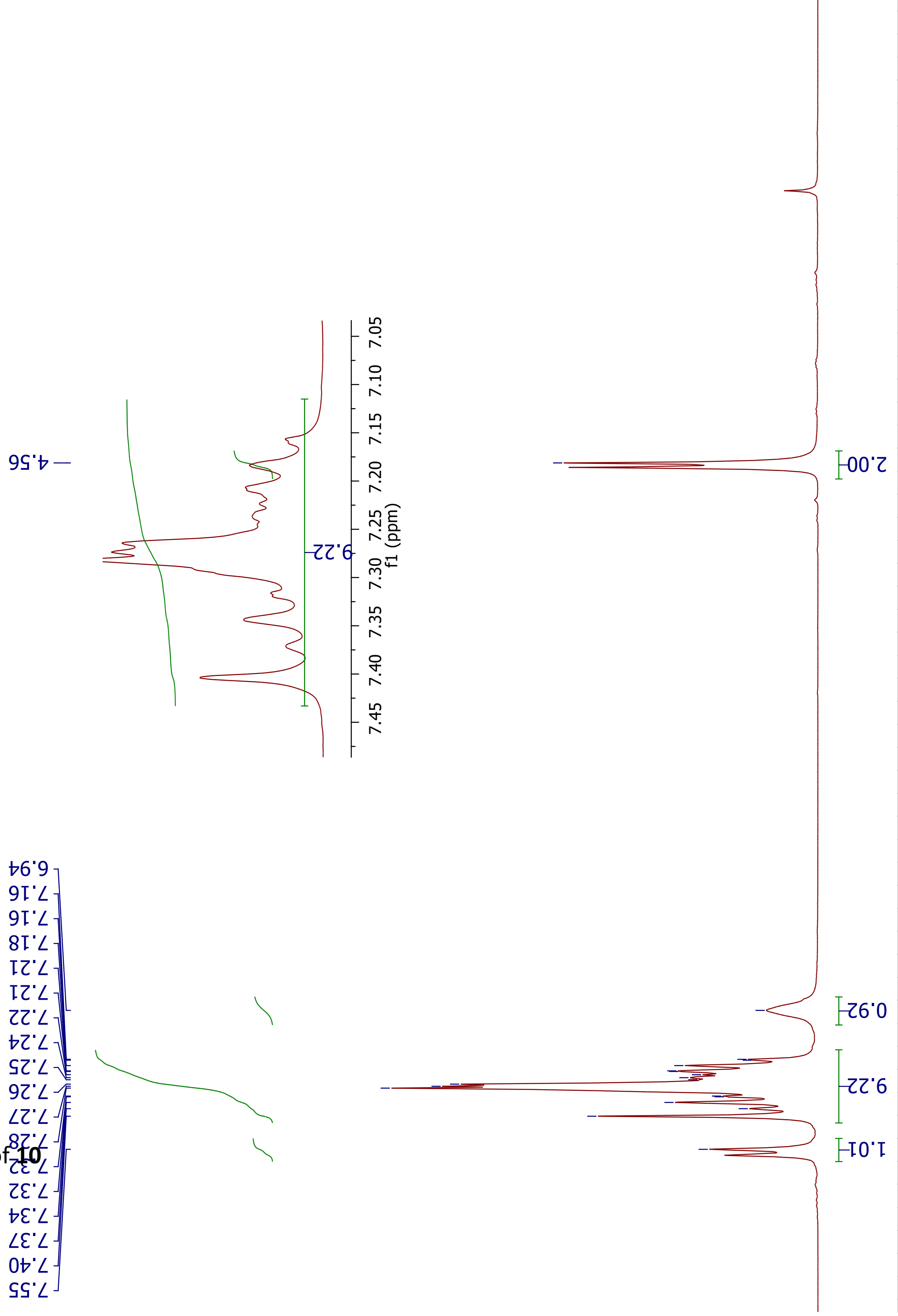


Figure S11 $^{1}$H-NMR of **10**

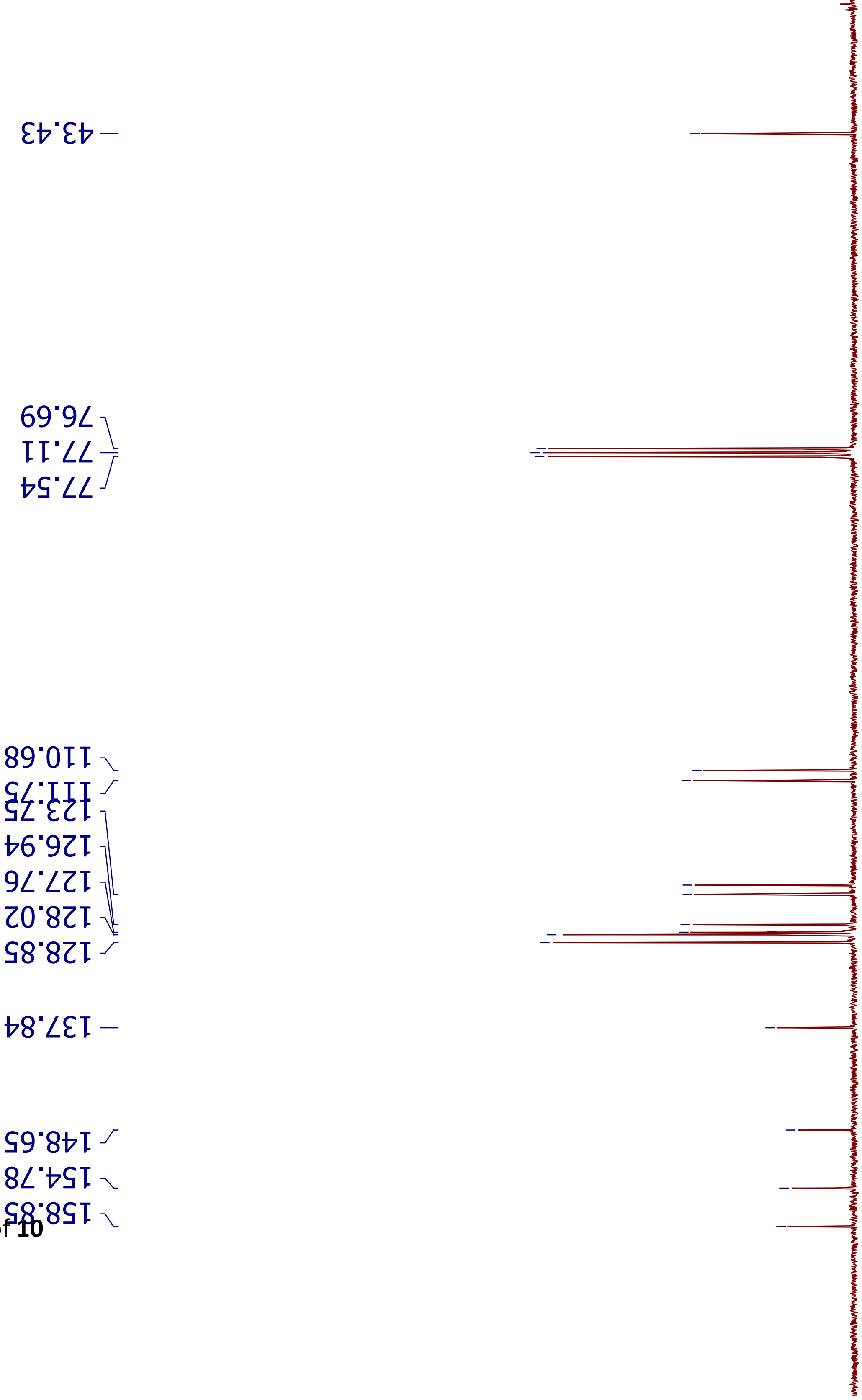


Figure S12 $^{13}$C-NMR of **10**

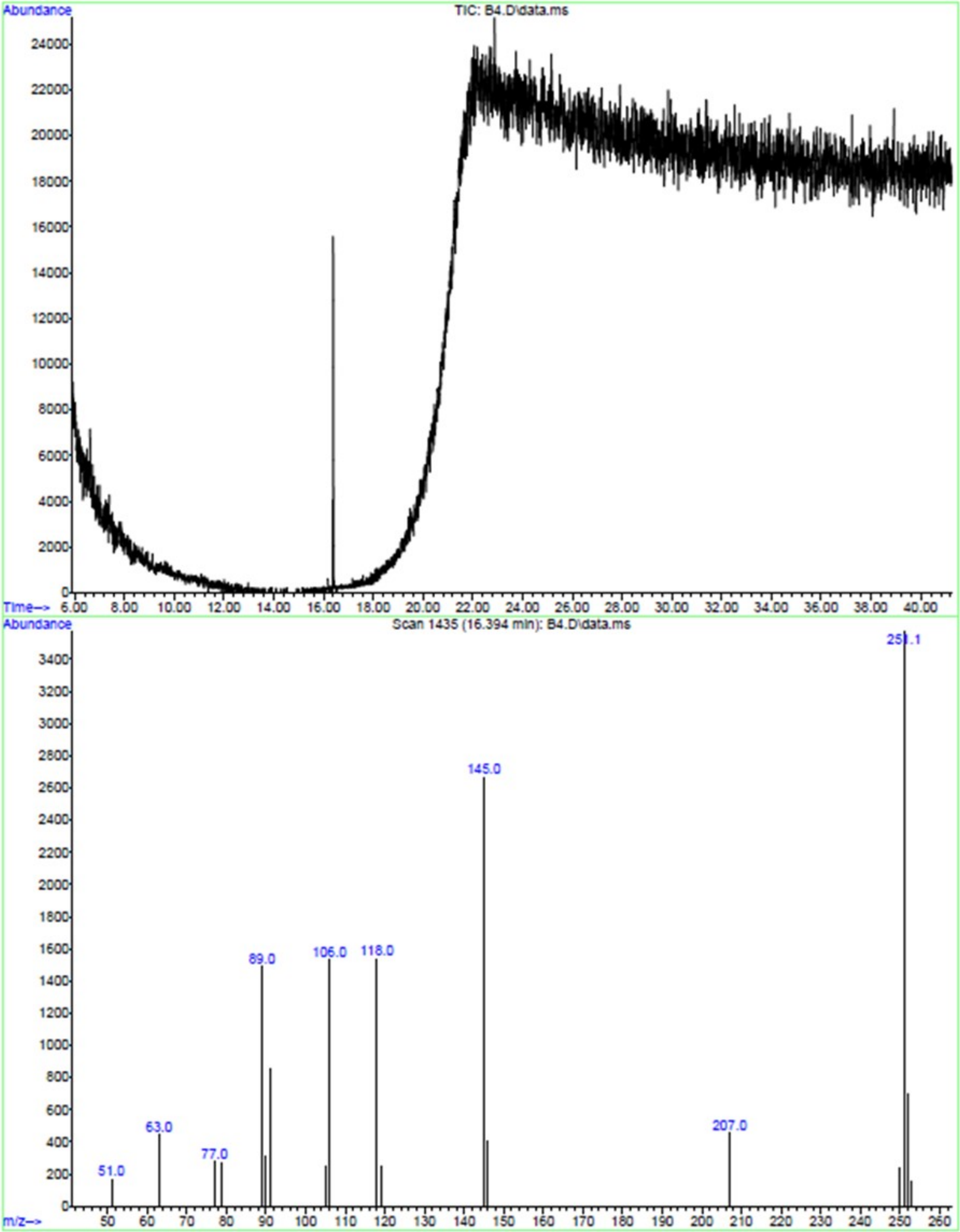


Figure S13 $^{13}$C-NMR of **10**

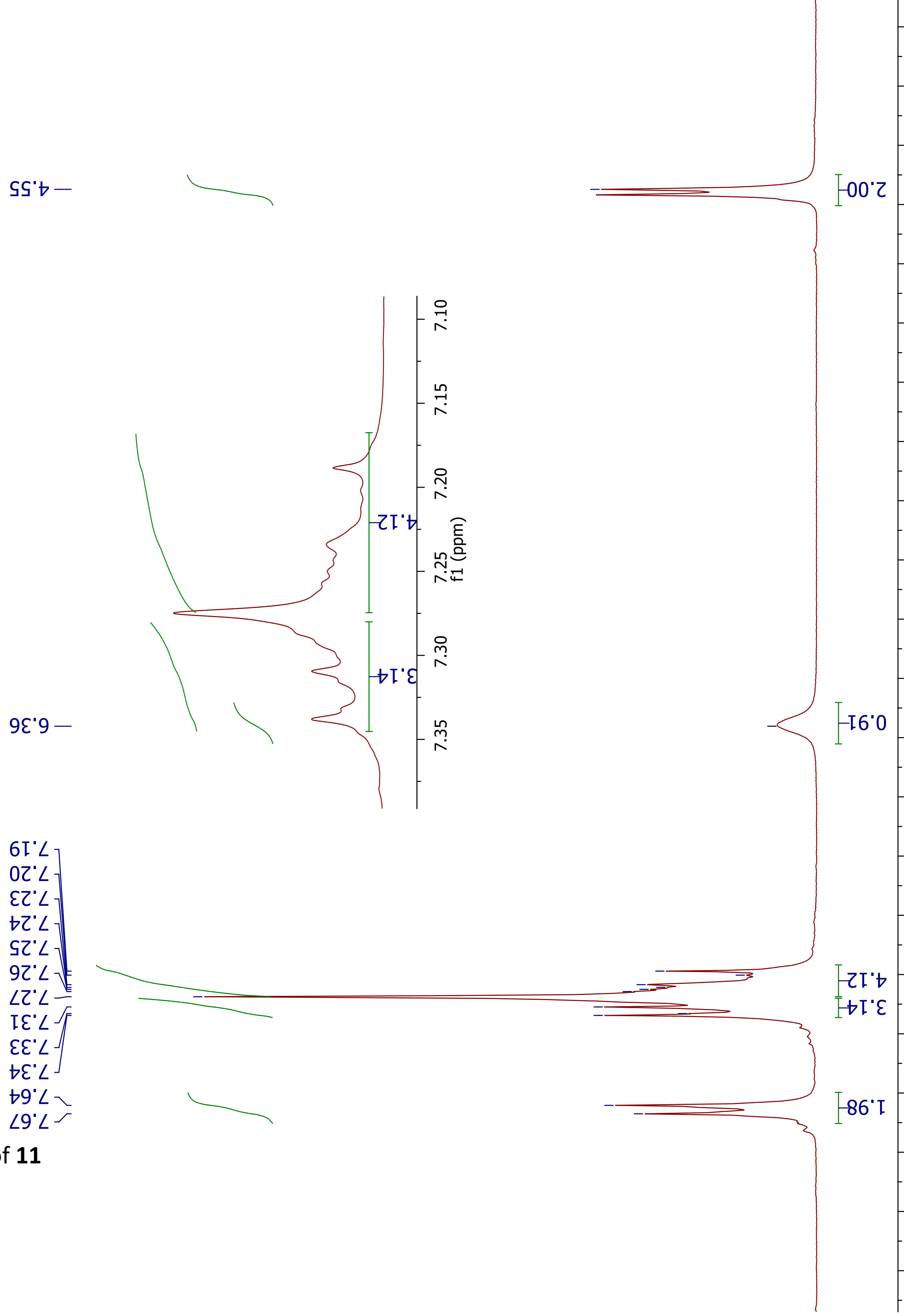


Figure S14 $^{1}$H NMR of **11**

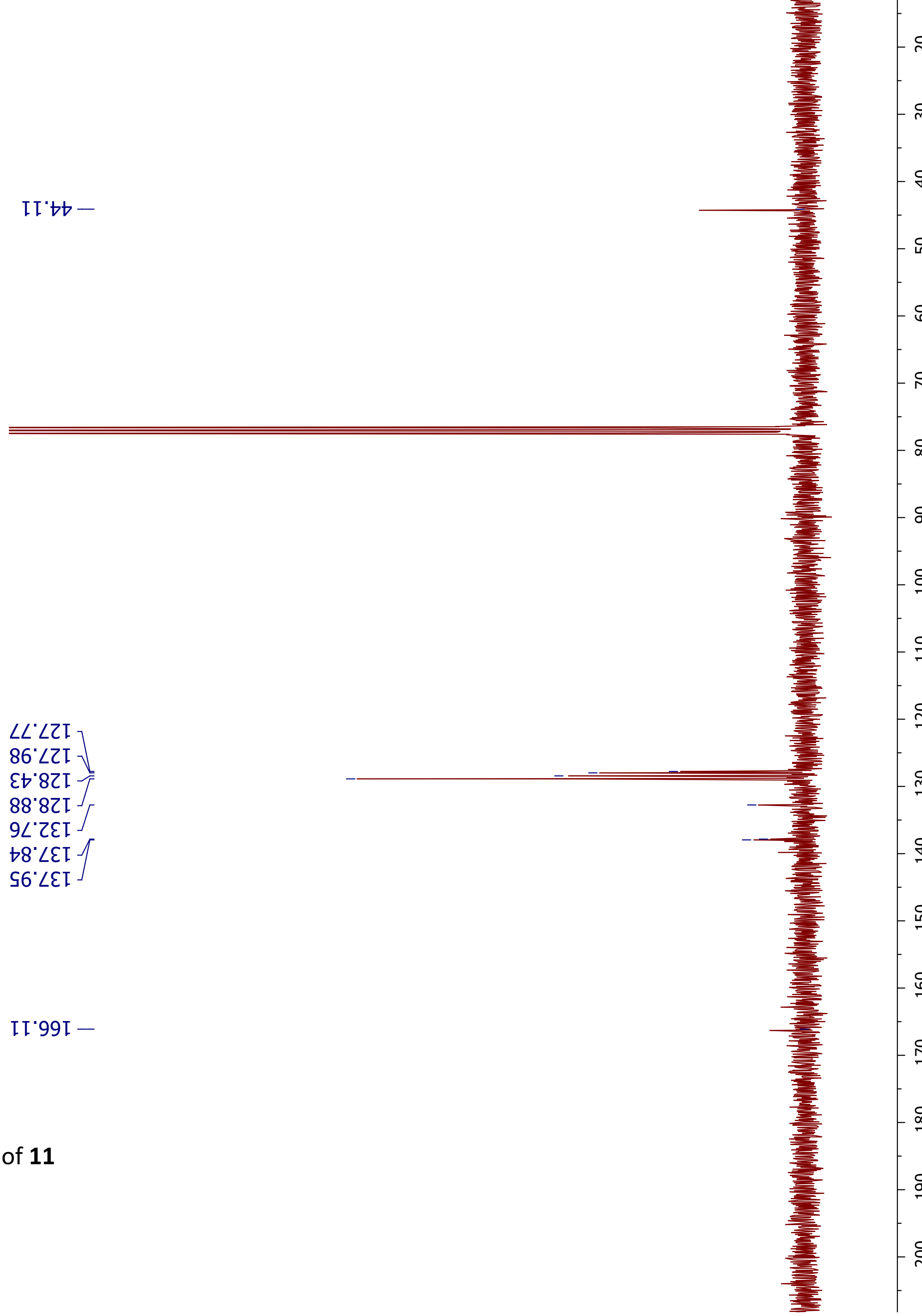


Figure S15 $^{13}C$-NMR of **11**

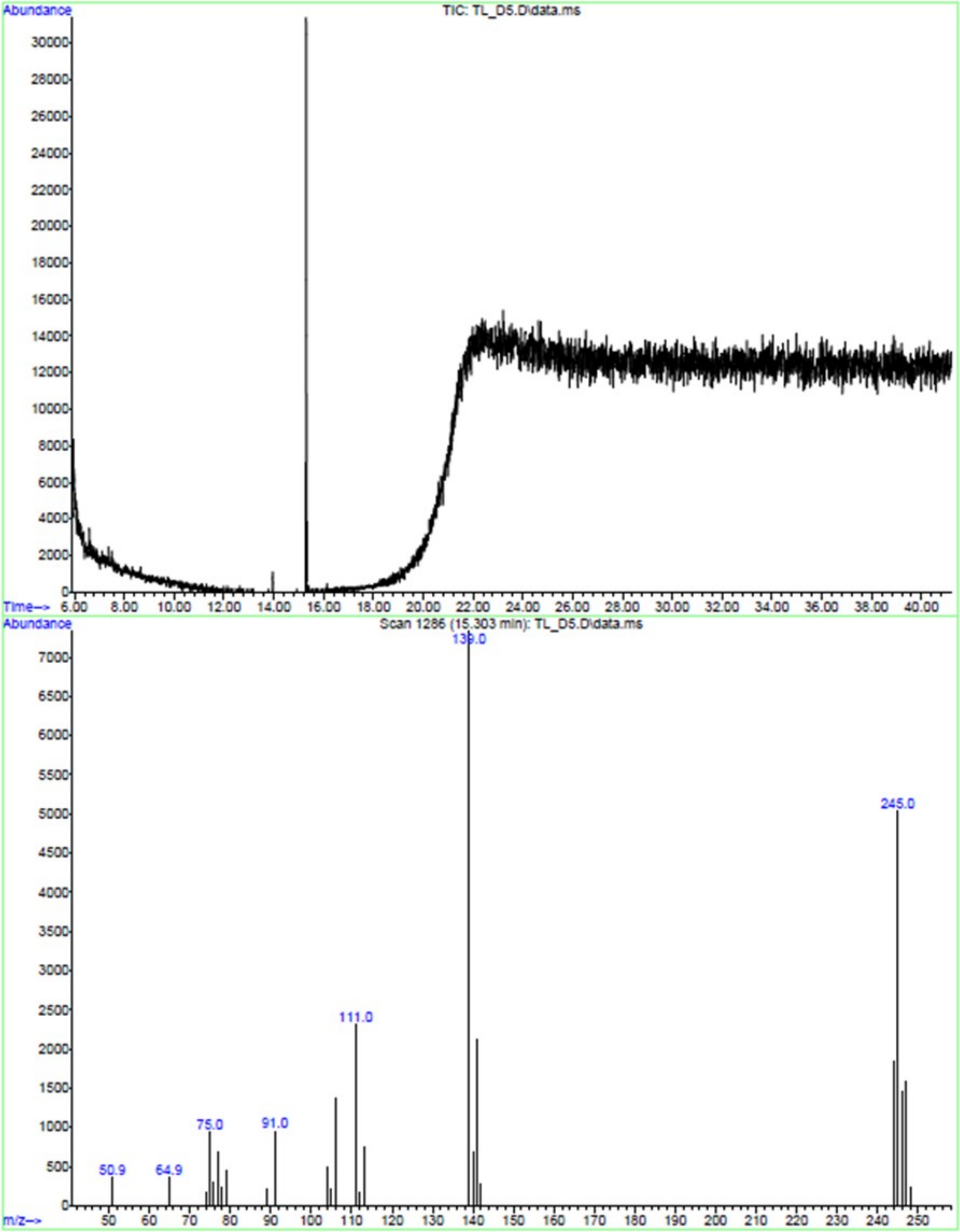


Figure S16 GC-MS of **11**

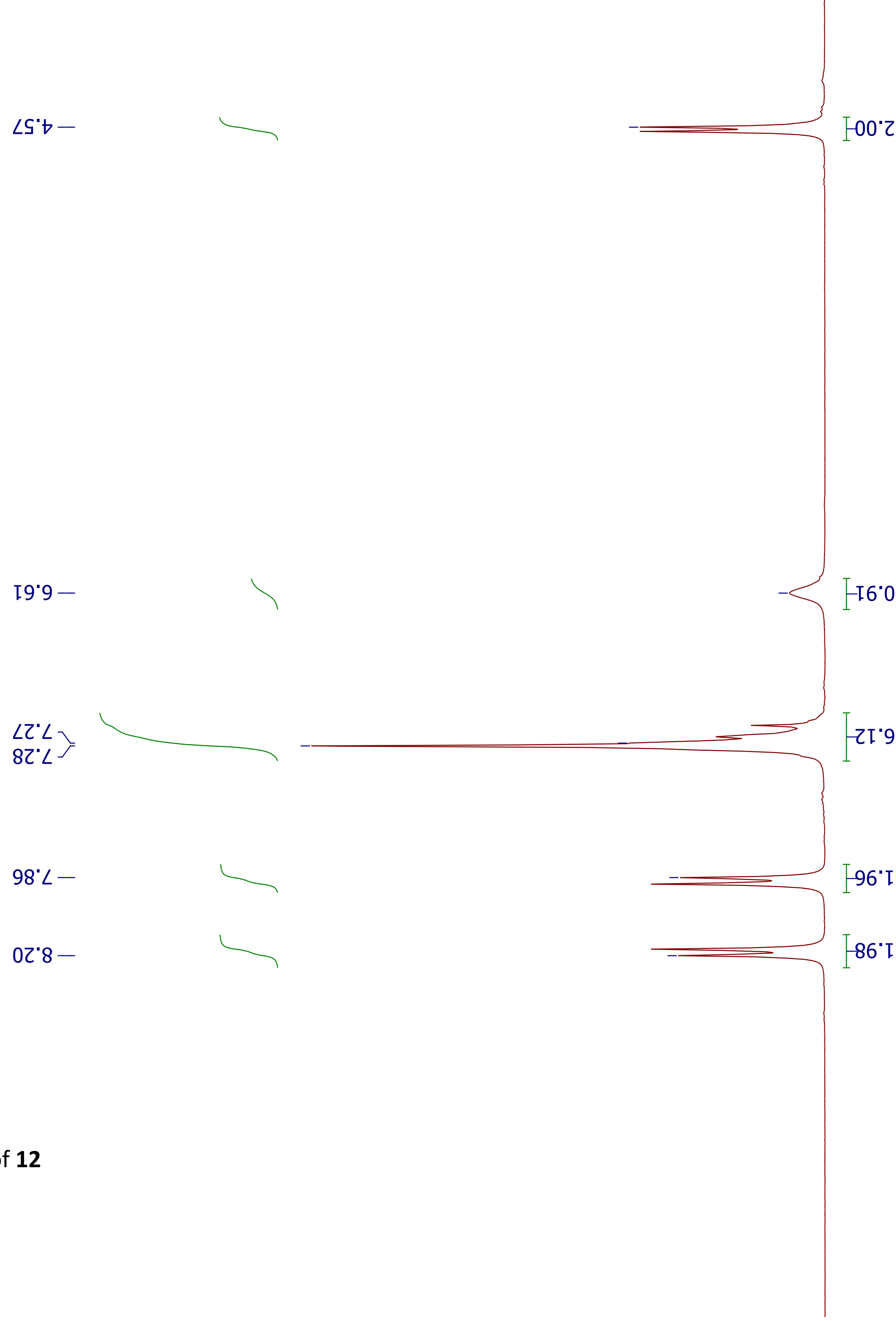


Figure S17 $^{1}$H NMR of **12**

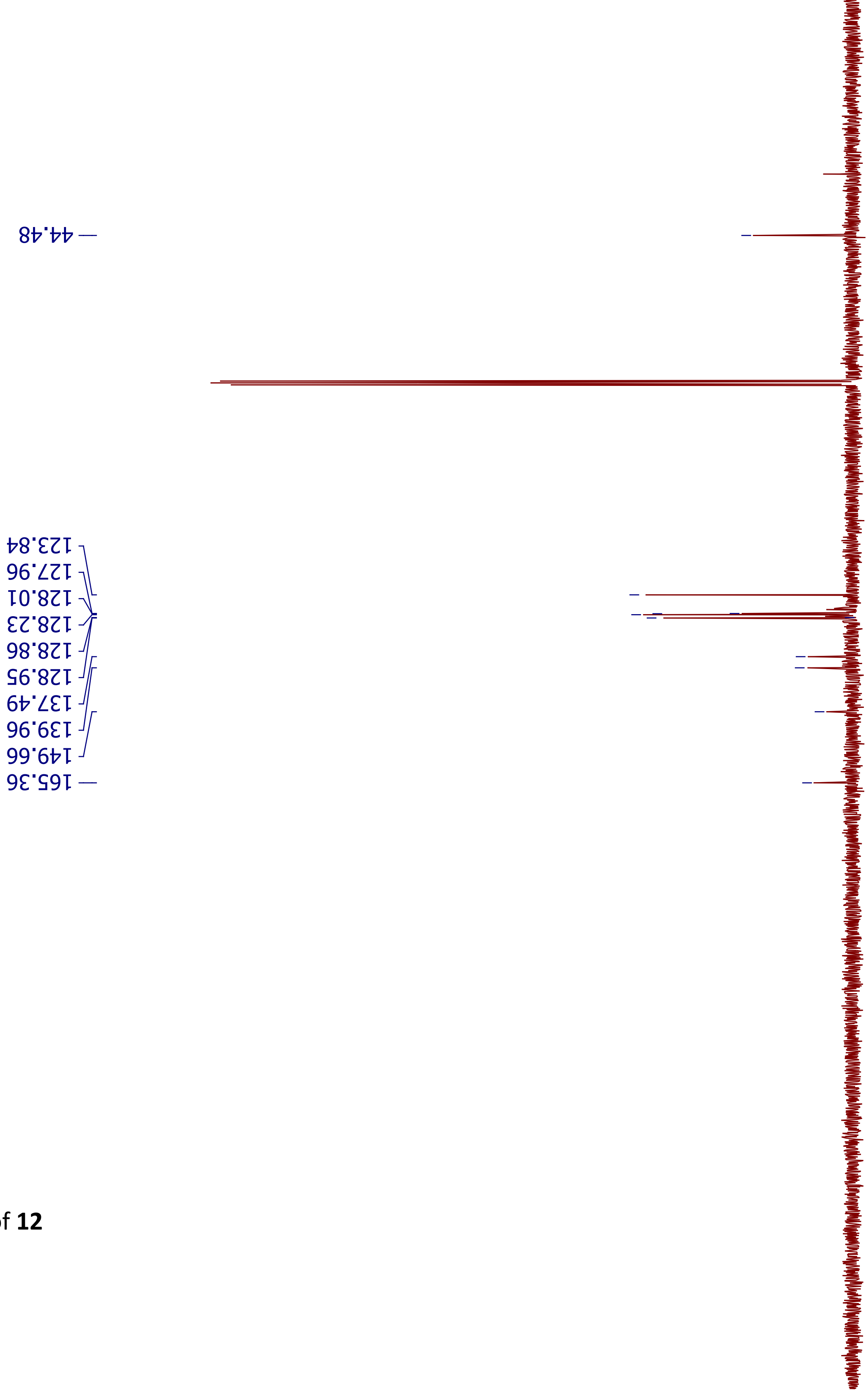


Figure S18 $^{13}$C-NMR of **12**

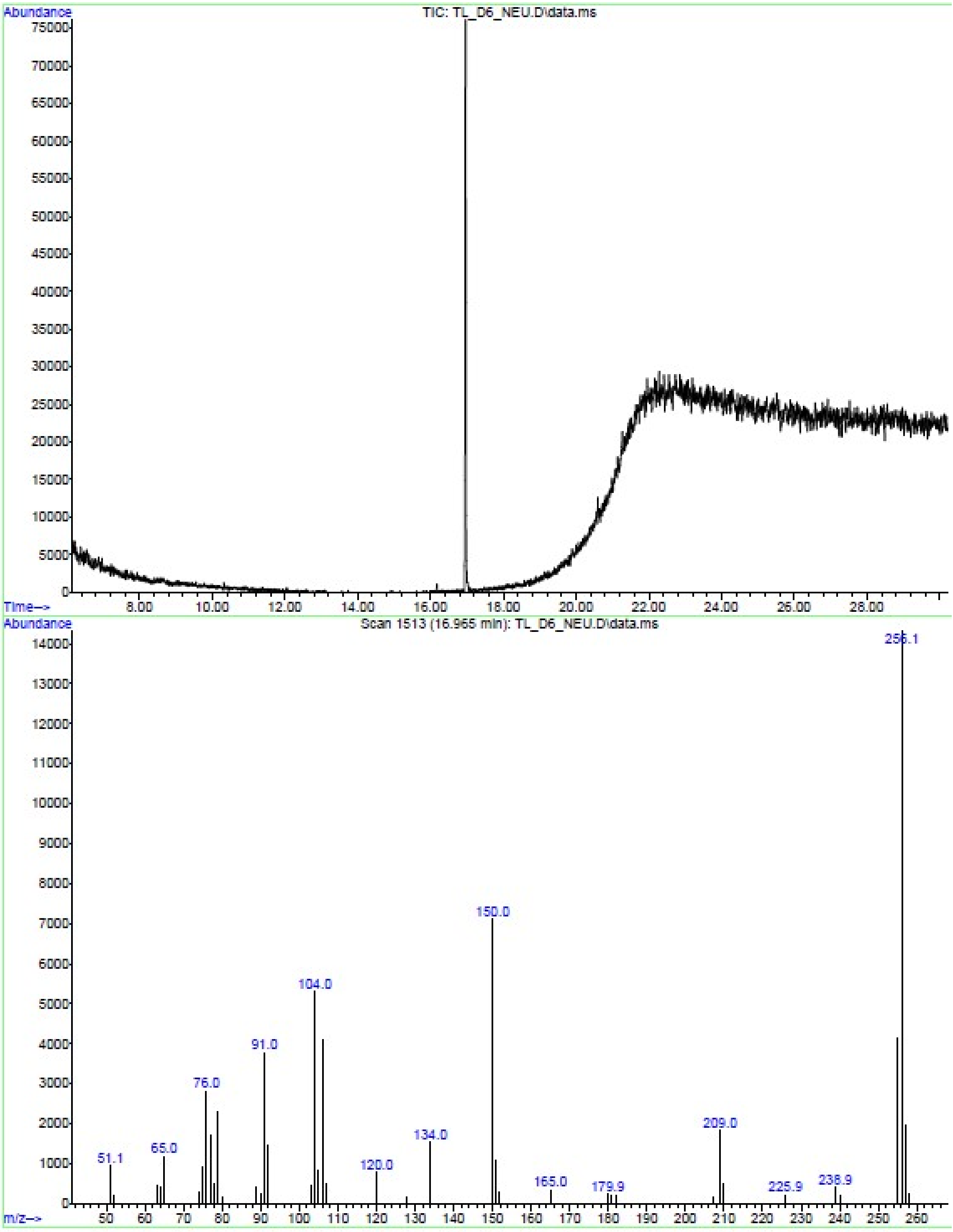


Figure S19 GC-MS of **12**

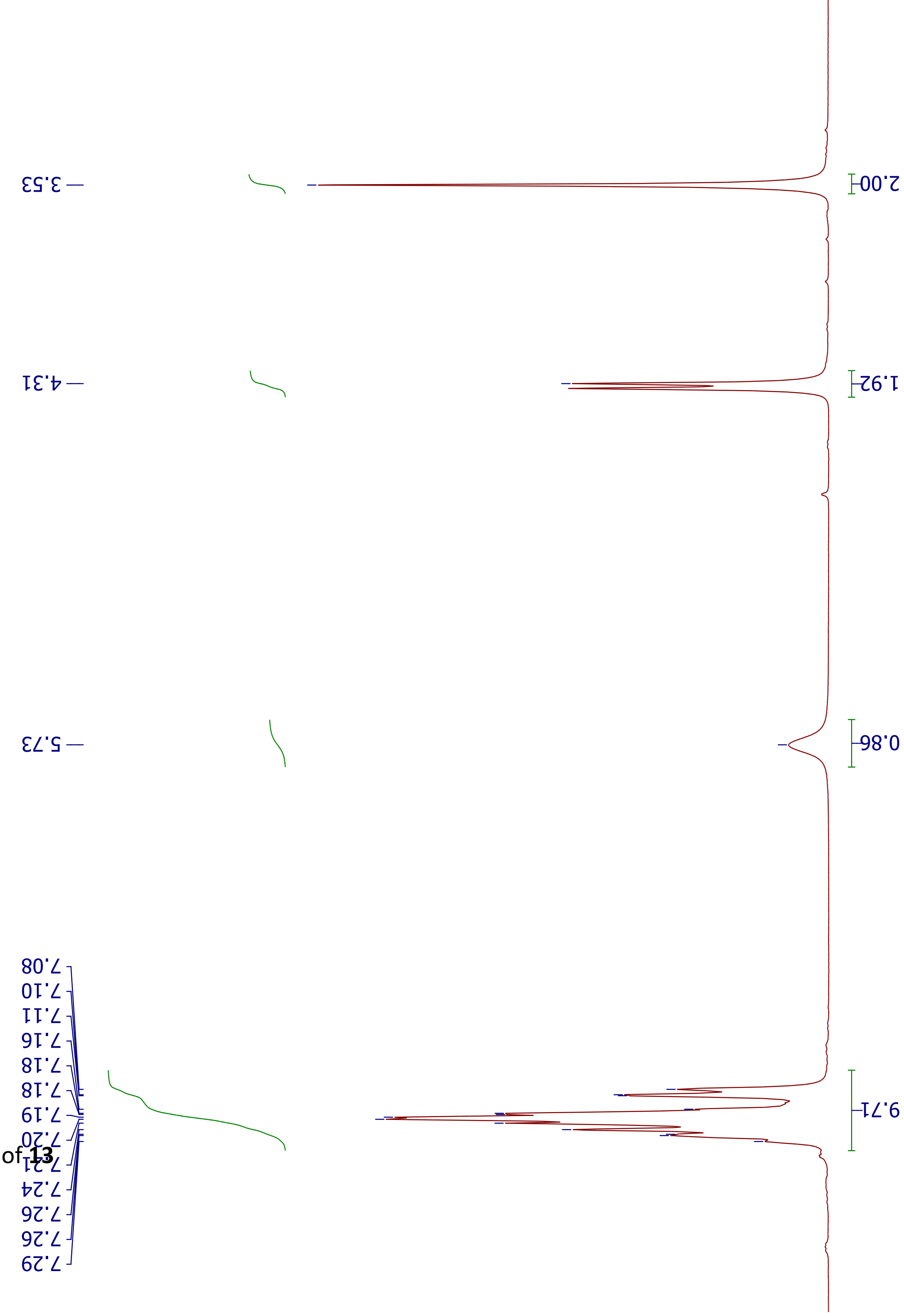


Figure S20 $^{1}$H-NMR of **13**

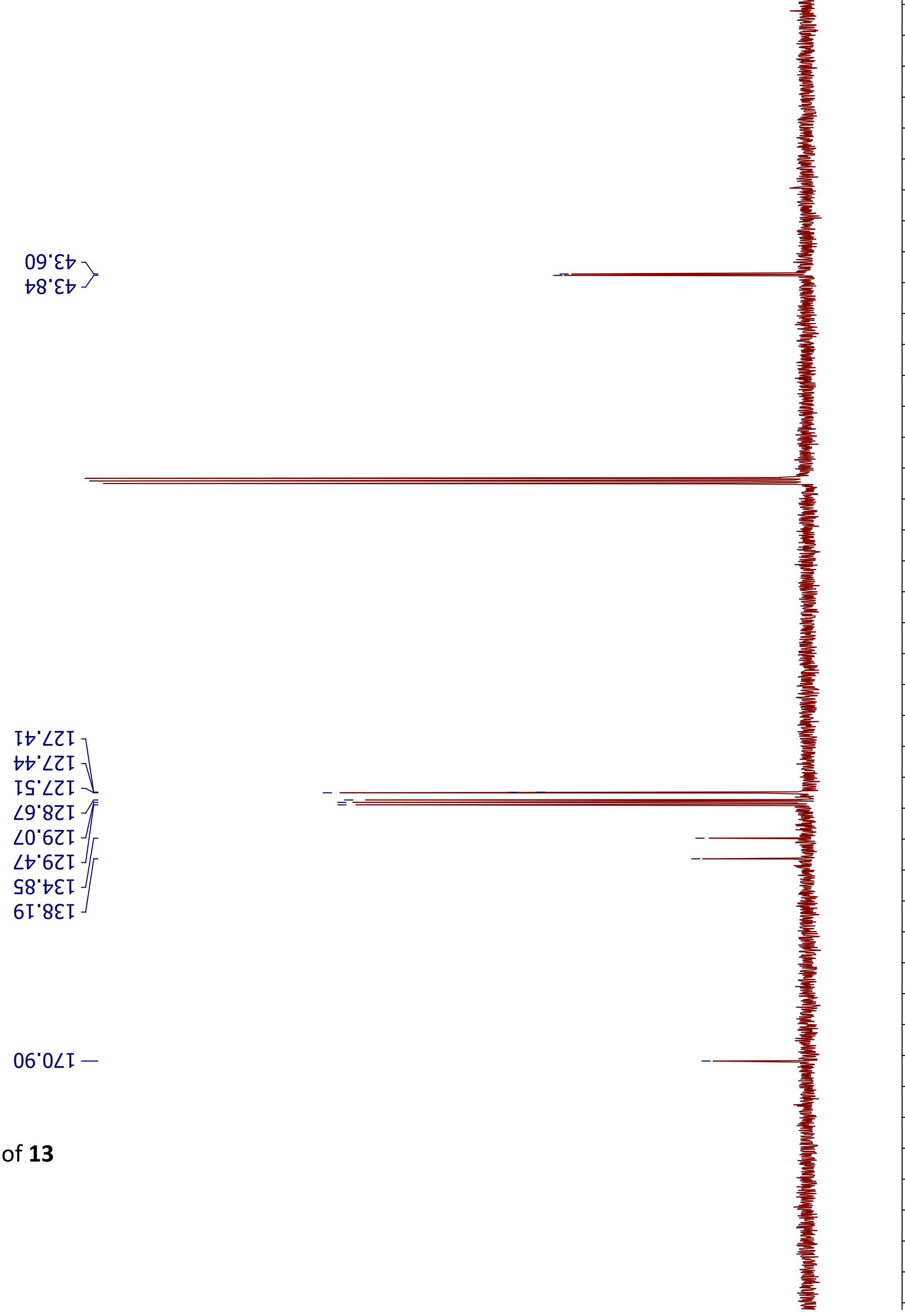


Figure S21 $^{13}$C-NMR of **13**

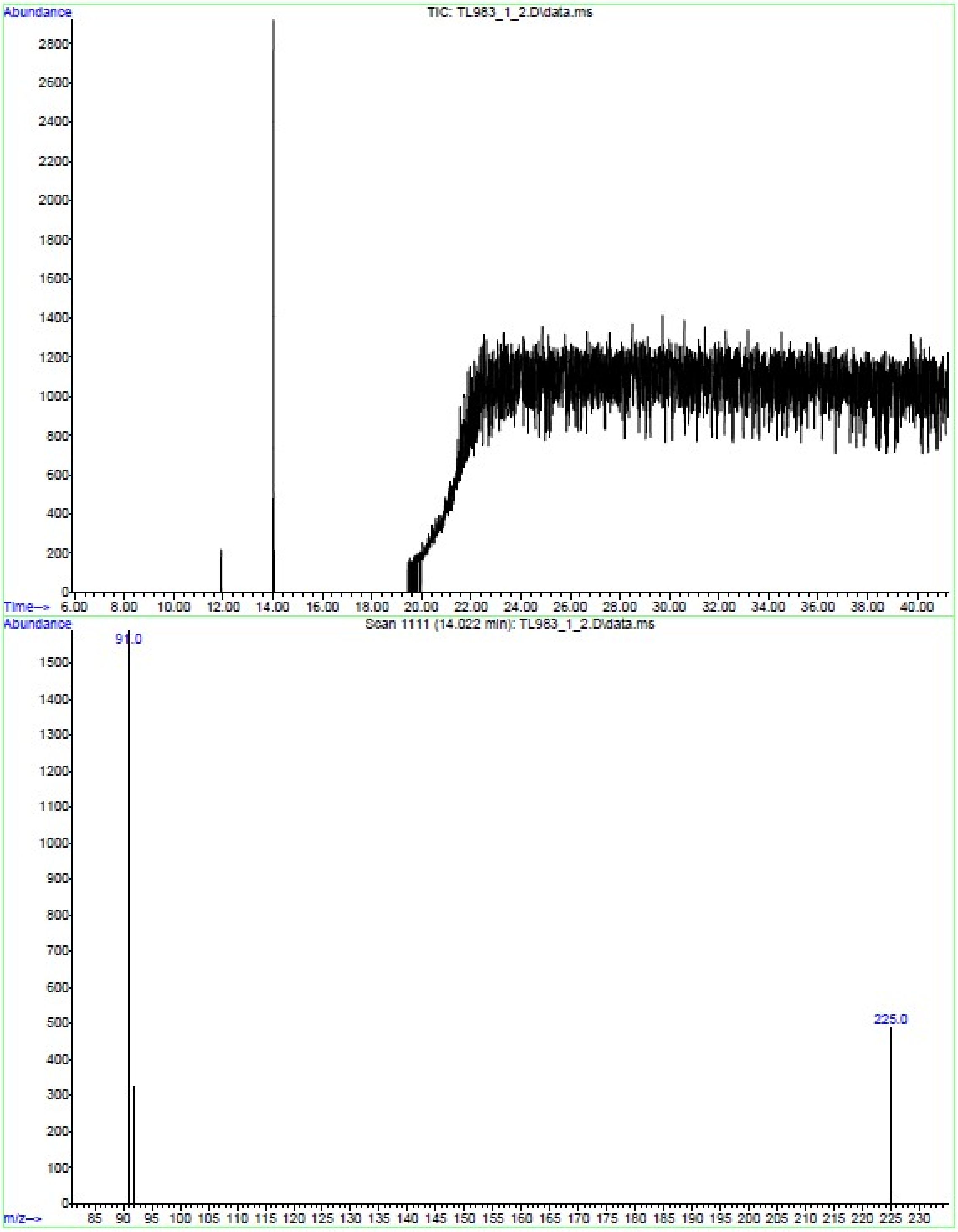


Figure S22 GC-MS of **13**

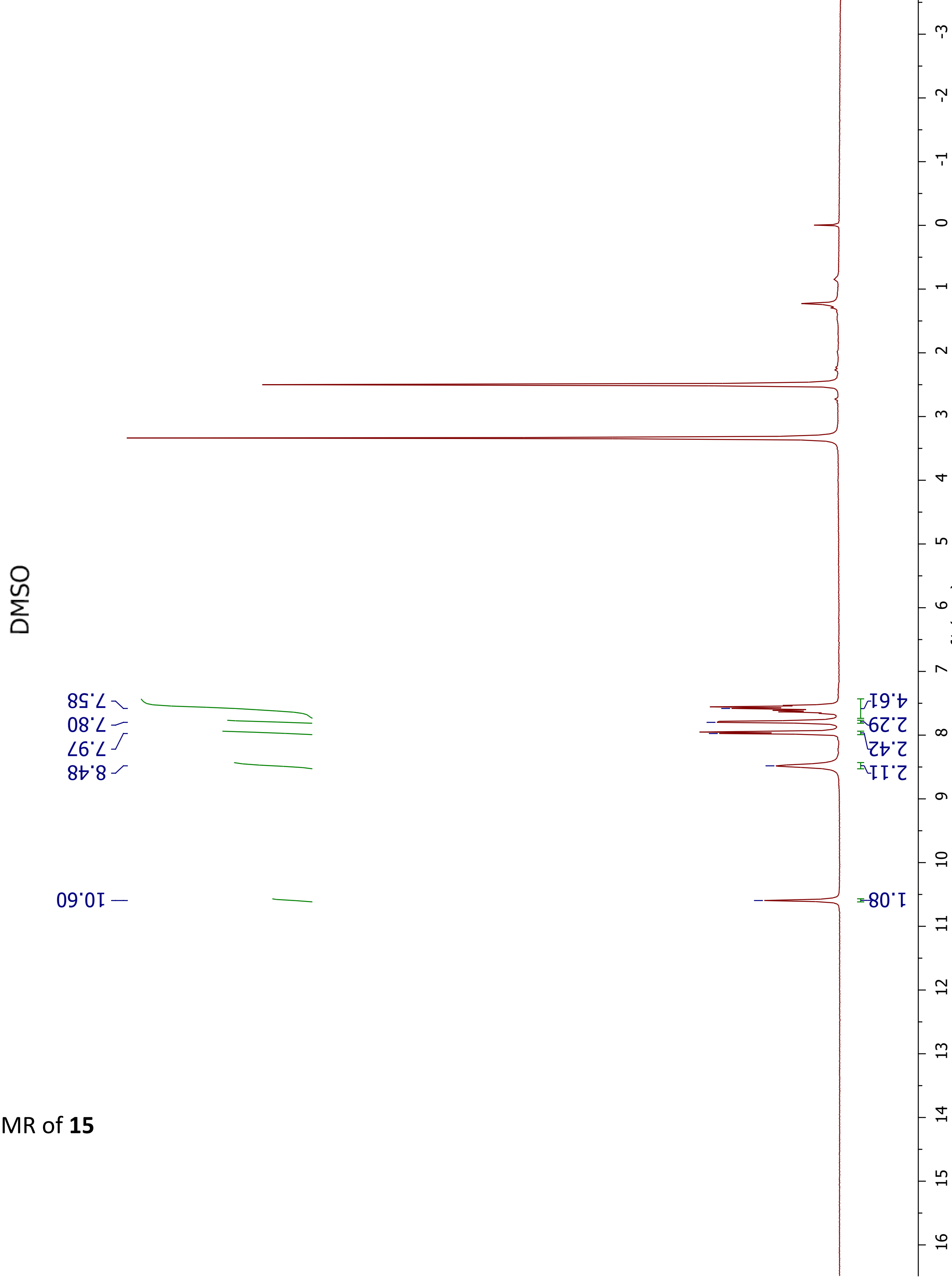


Figure S23 $^1H$ NMR of **15**

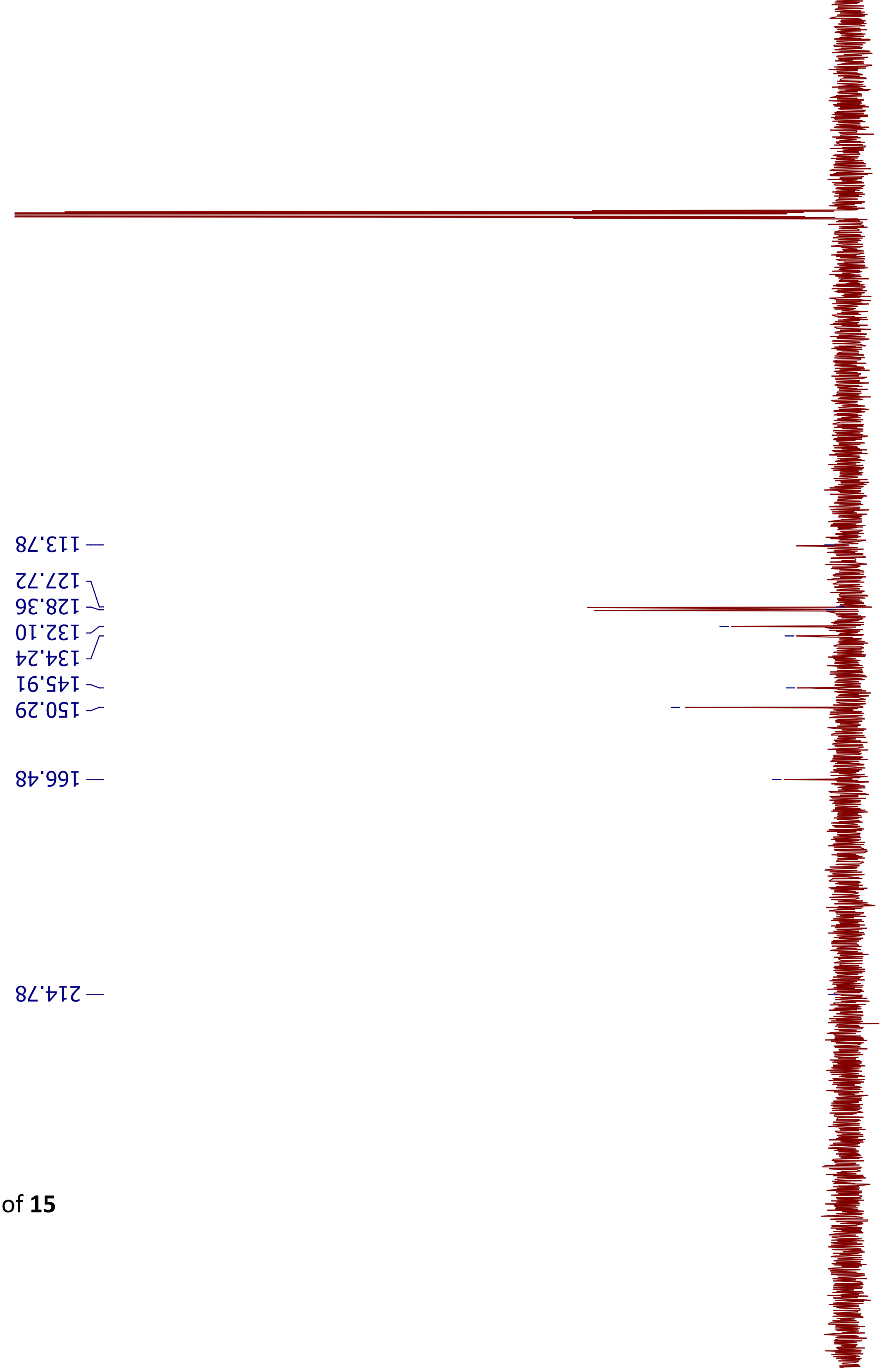


Figure S24 $^{13}$C-NMR of **15**

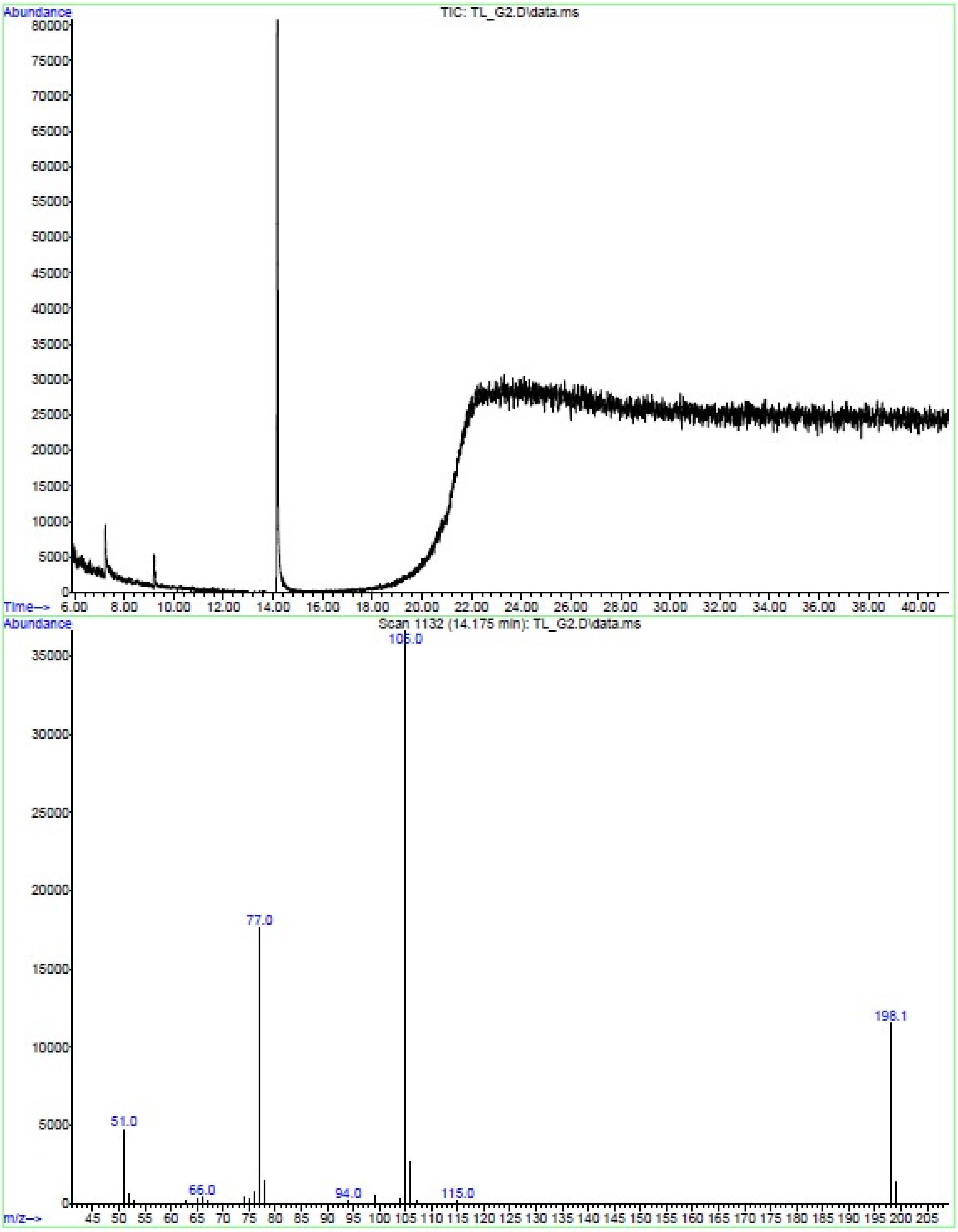


Figure S25 GC-MS of **15**

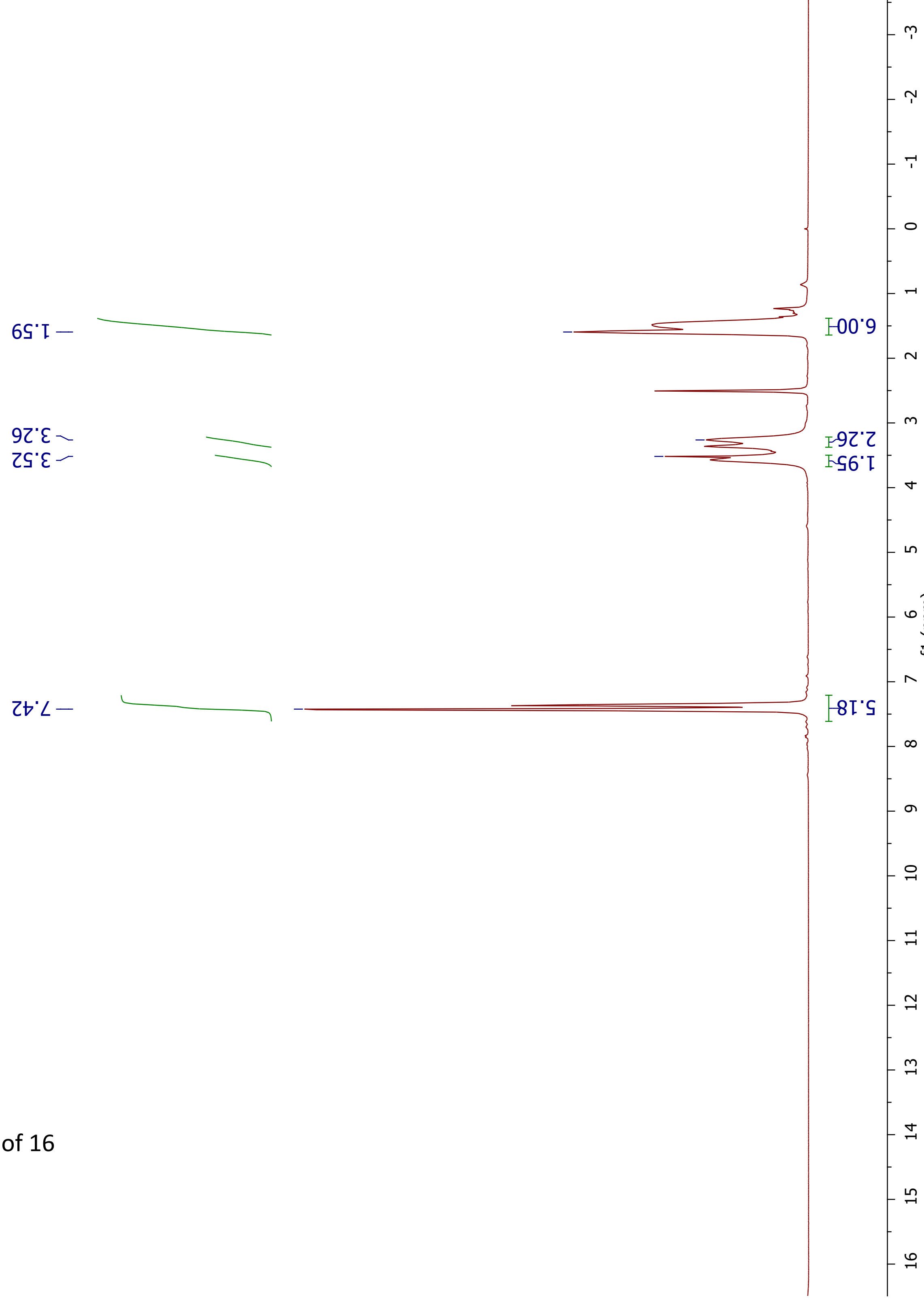


Figure S26 1H NMR of 16

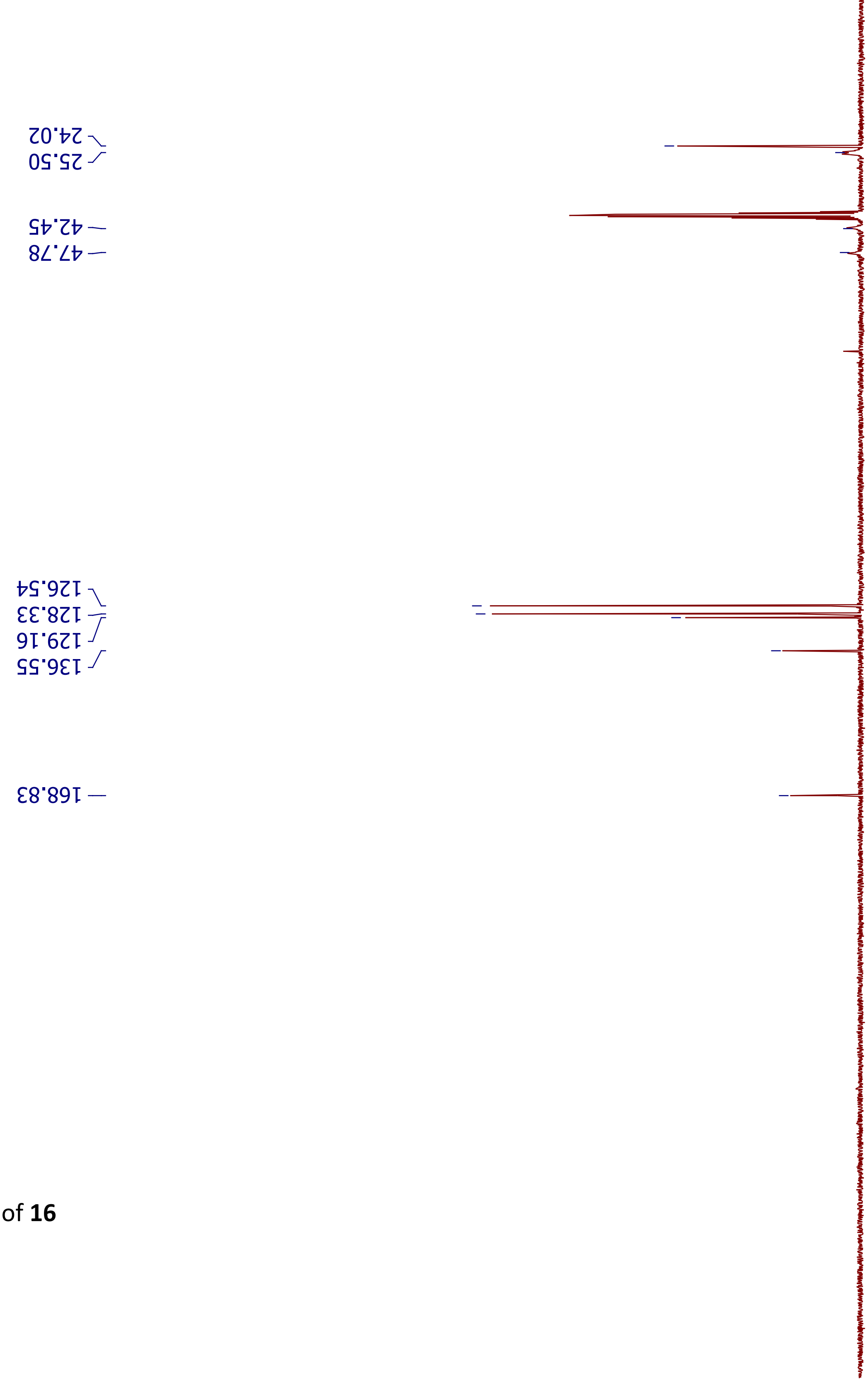


Figure S27 $^{13}$C-NMR of **16**

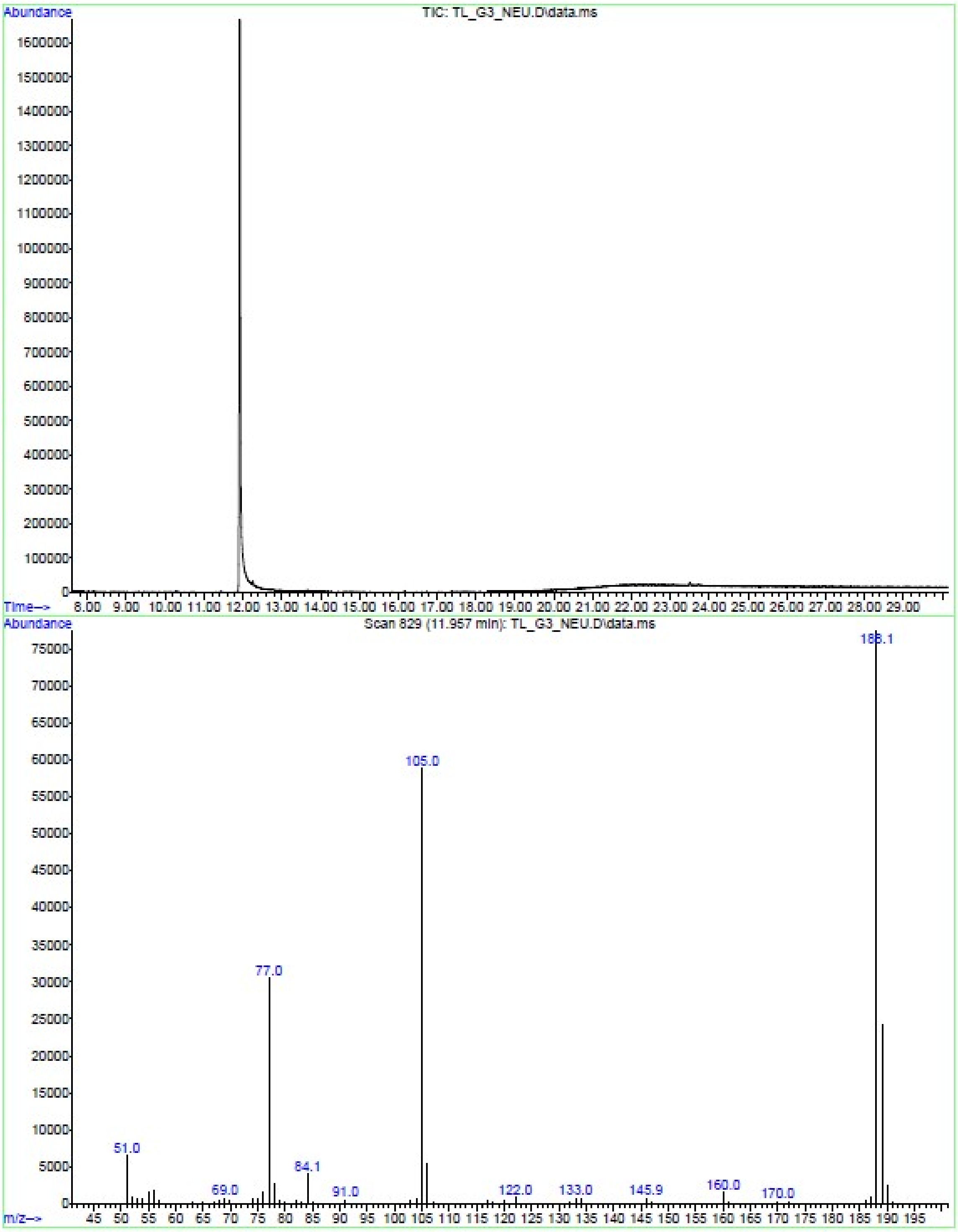


Figure S28 GC-MS of **16**

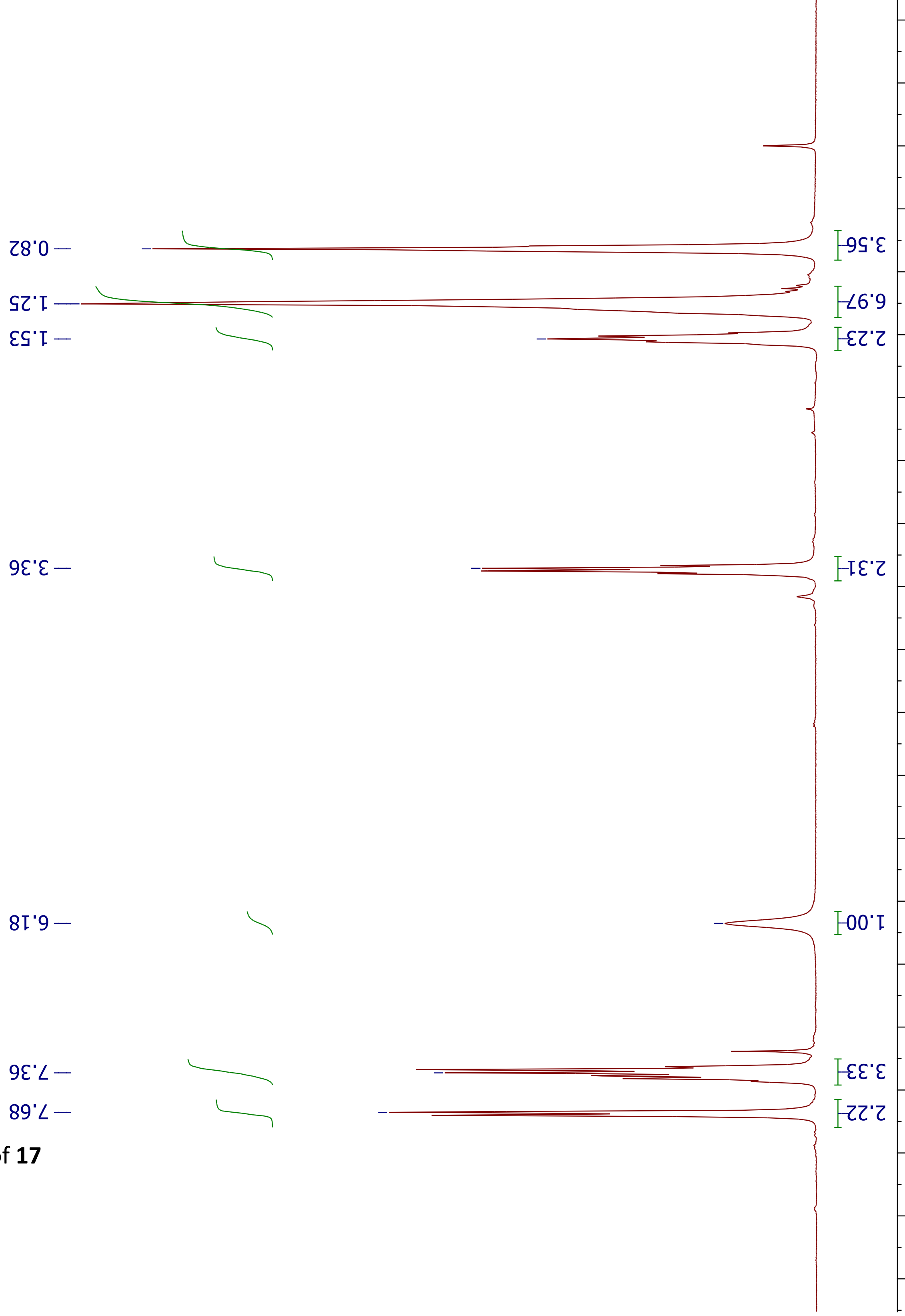


Figure S29 $^{1}H$ NMR of **17**

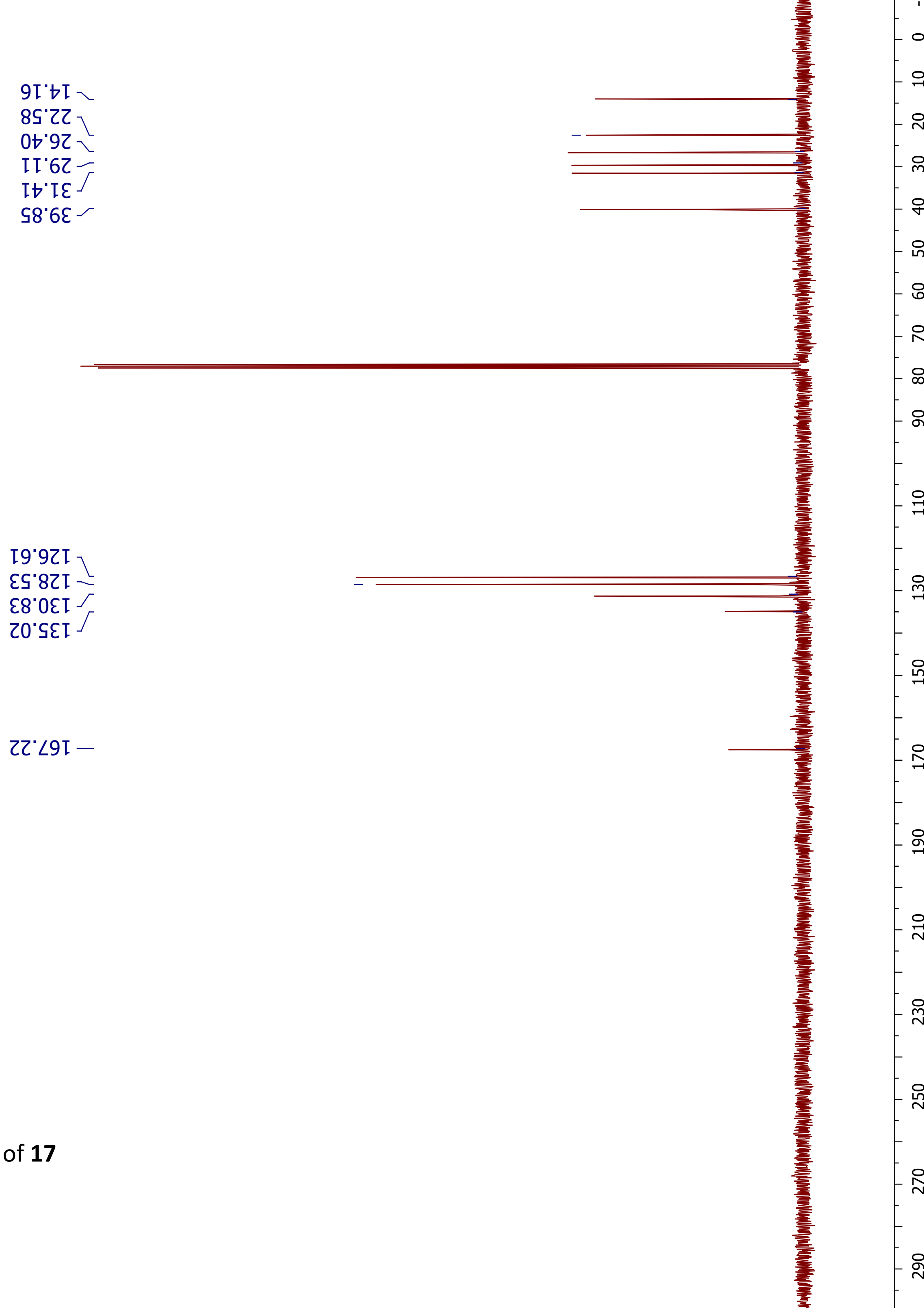


Figure S30 $^{13}$C-NMR of **17**

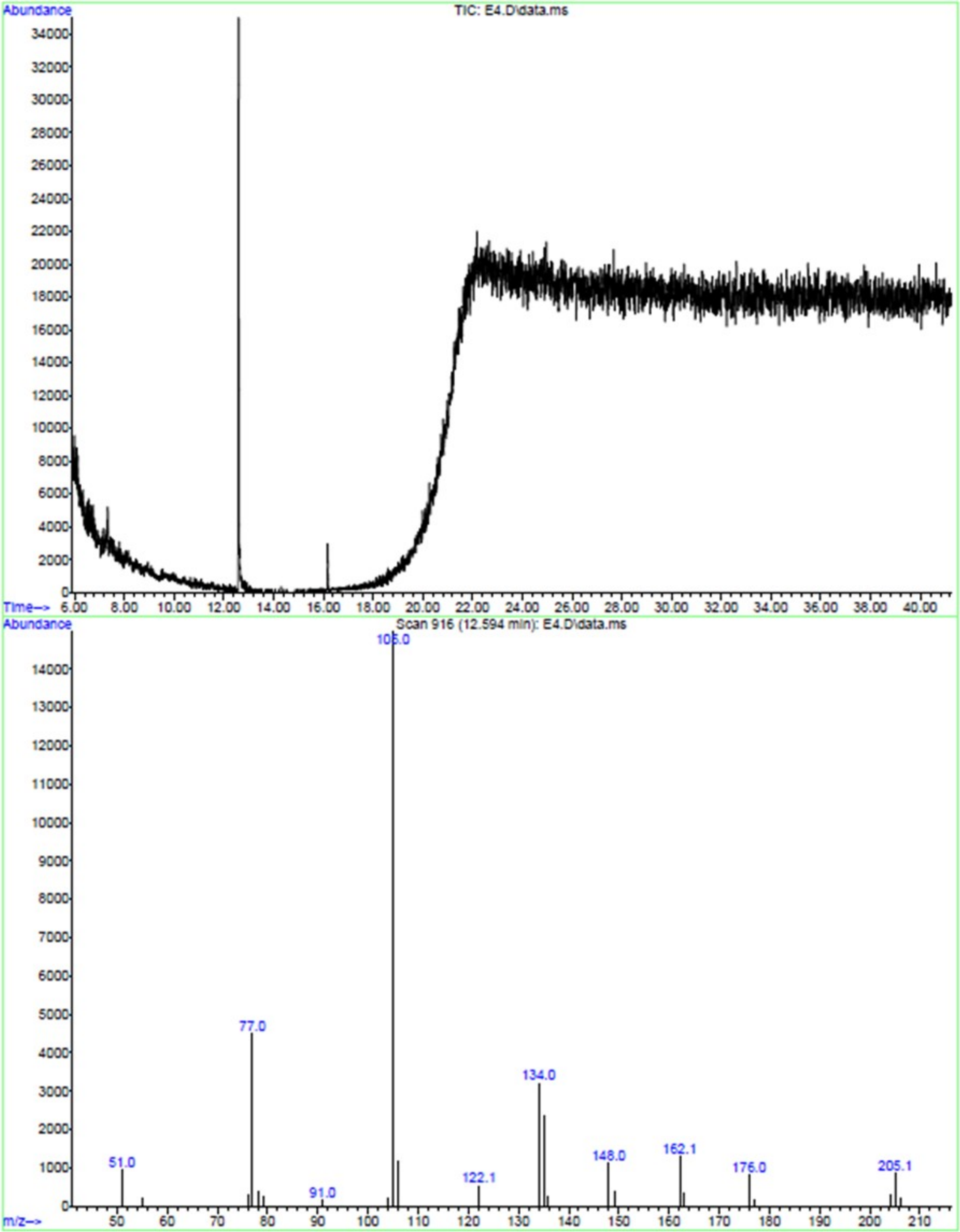


Figure S31 GC-MS of **17**

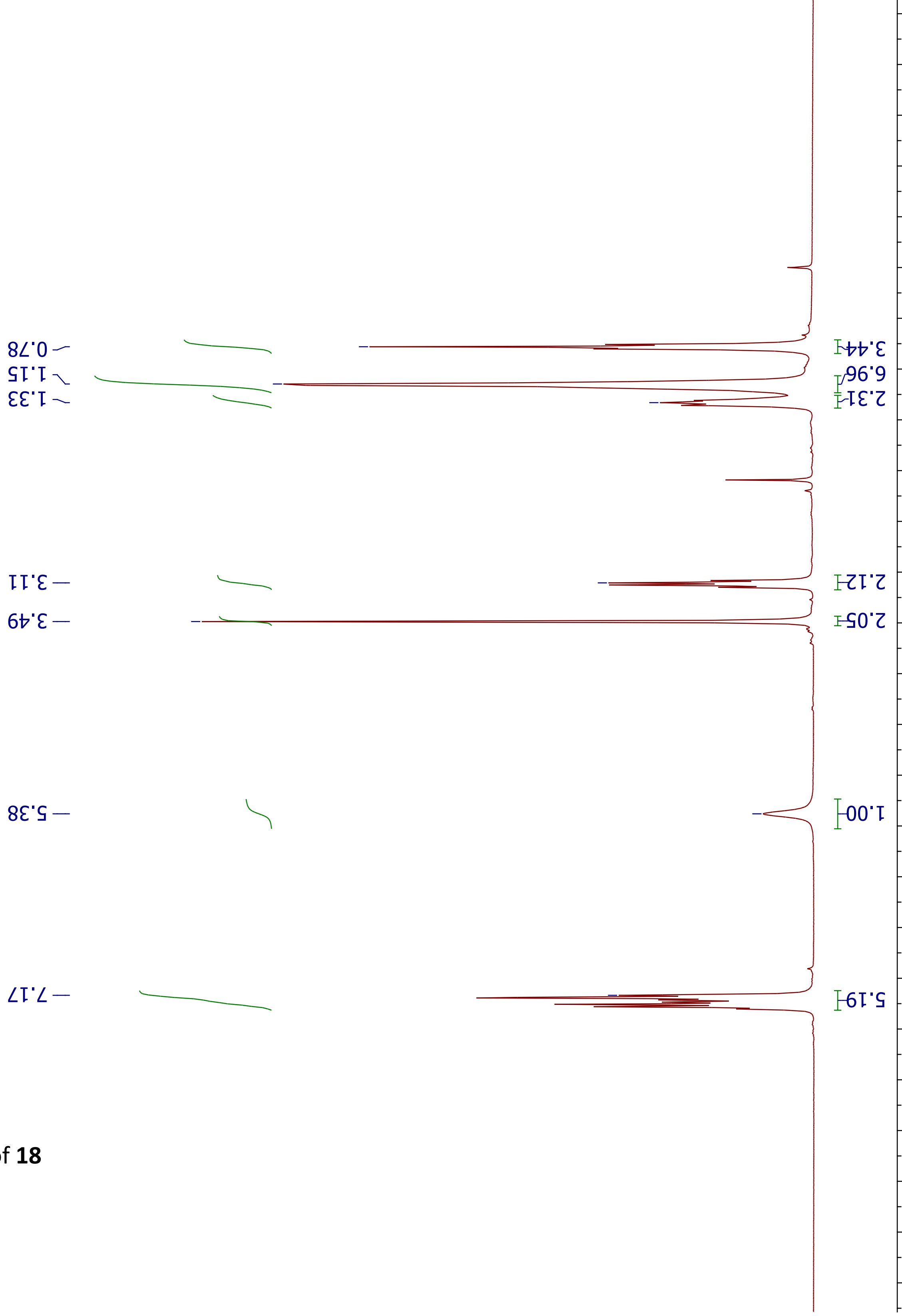


Figure S32 $^{1}$H-NMR of **18**

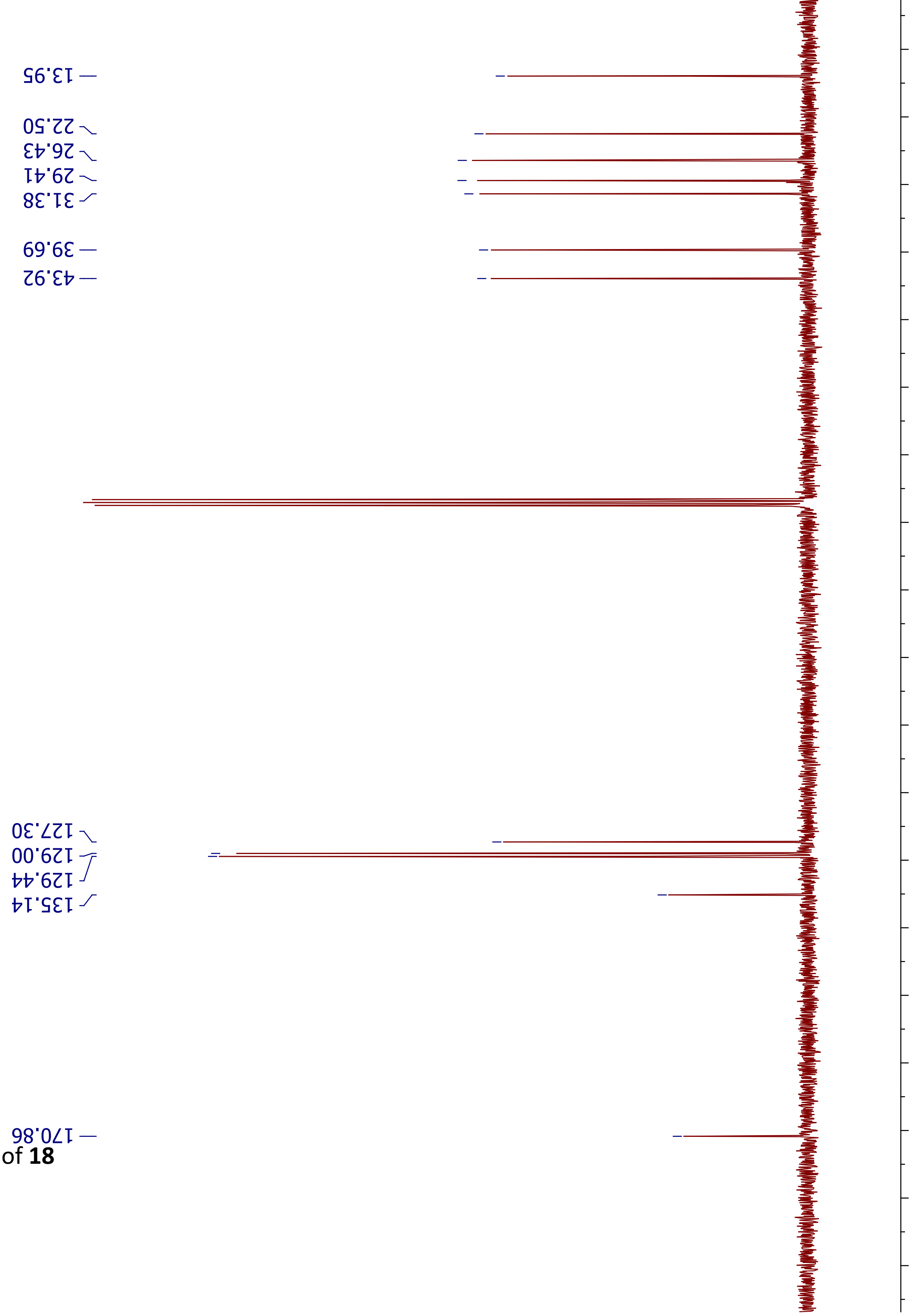


Figure S33 $^{13}$C-NMR of **18**

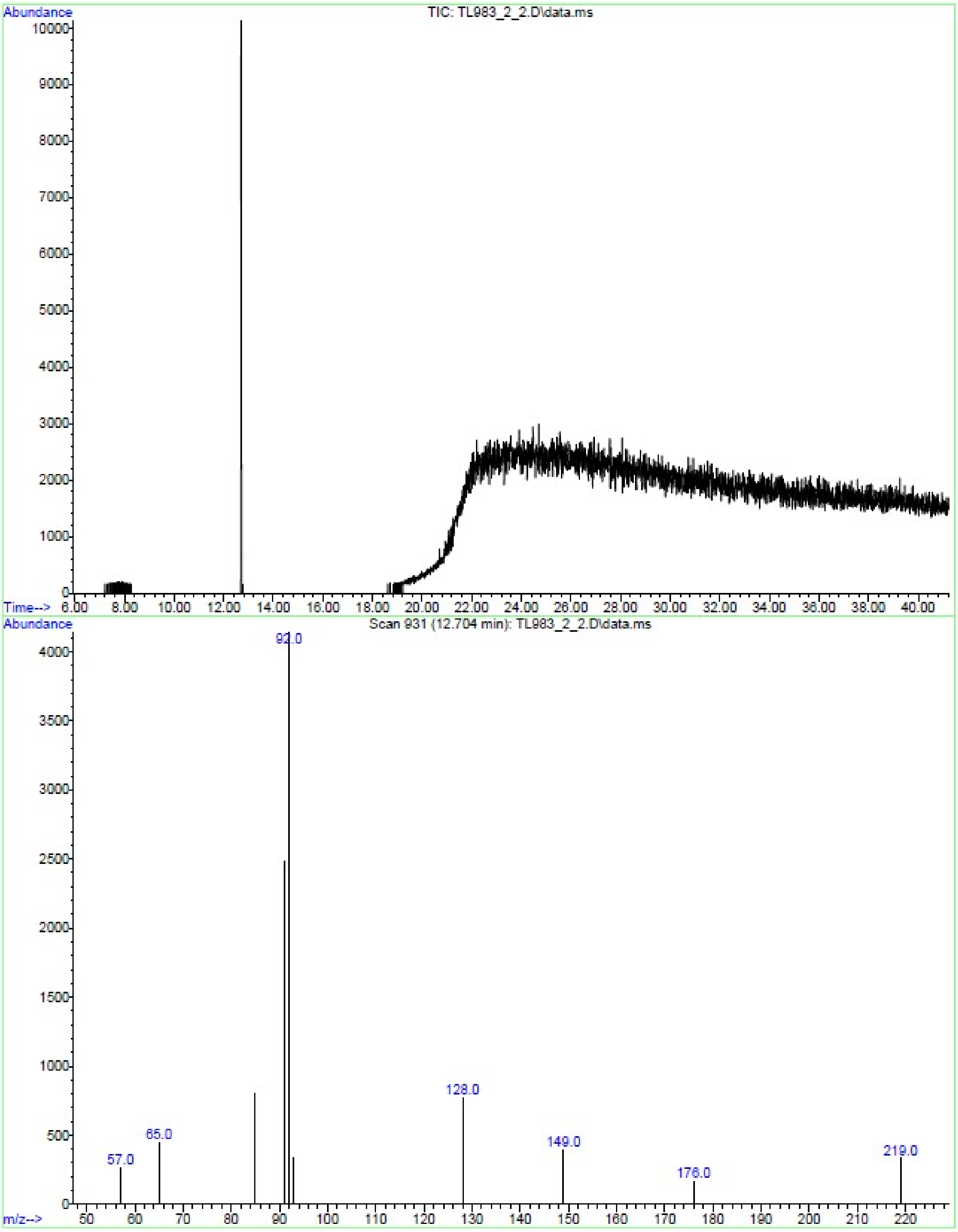


Figure S34 GC-MS of **18**

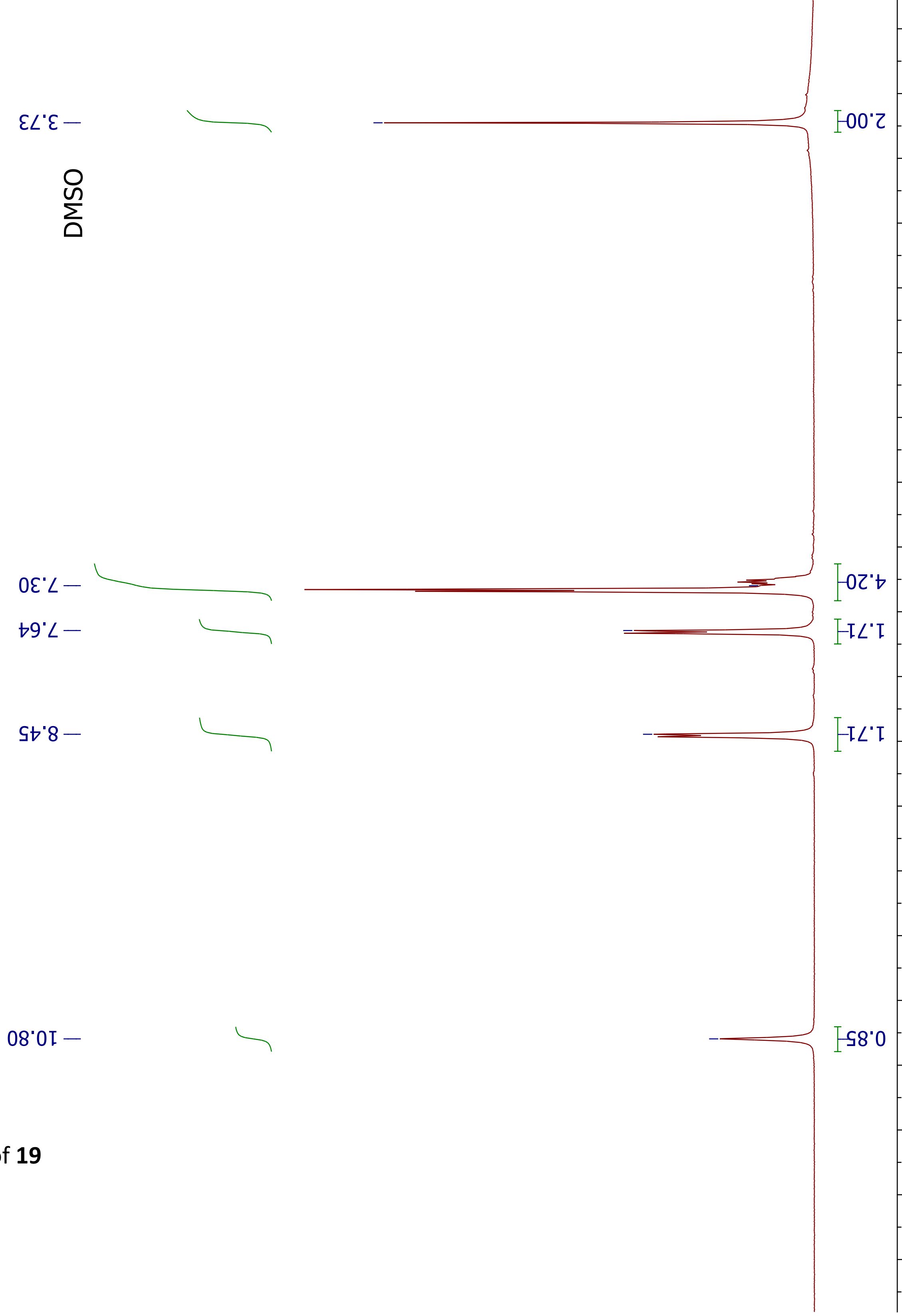


Figure S35 $^1$H-NMR of **19**

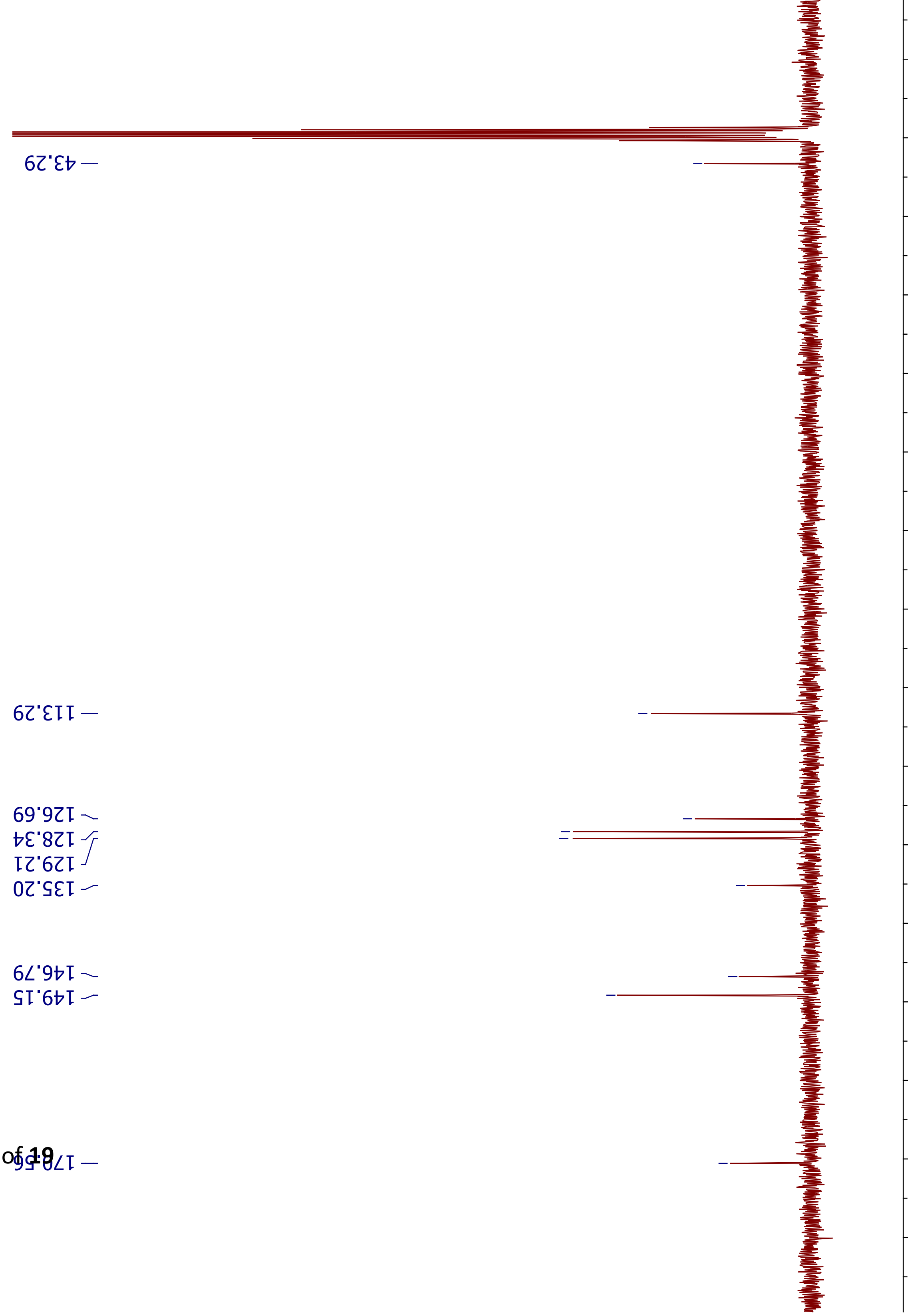


Figure S36 $^{13}$C-NMR of **19**

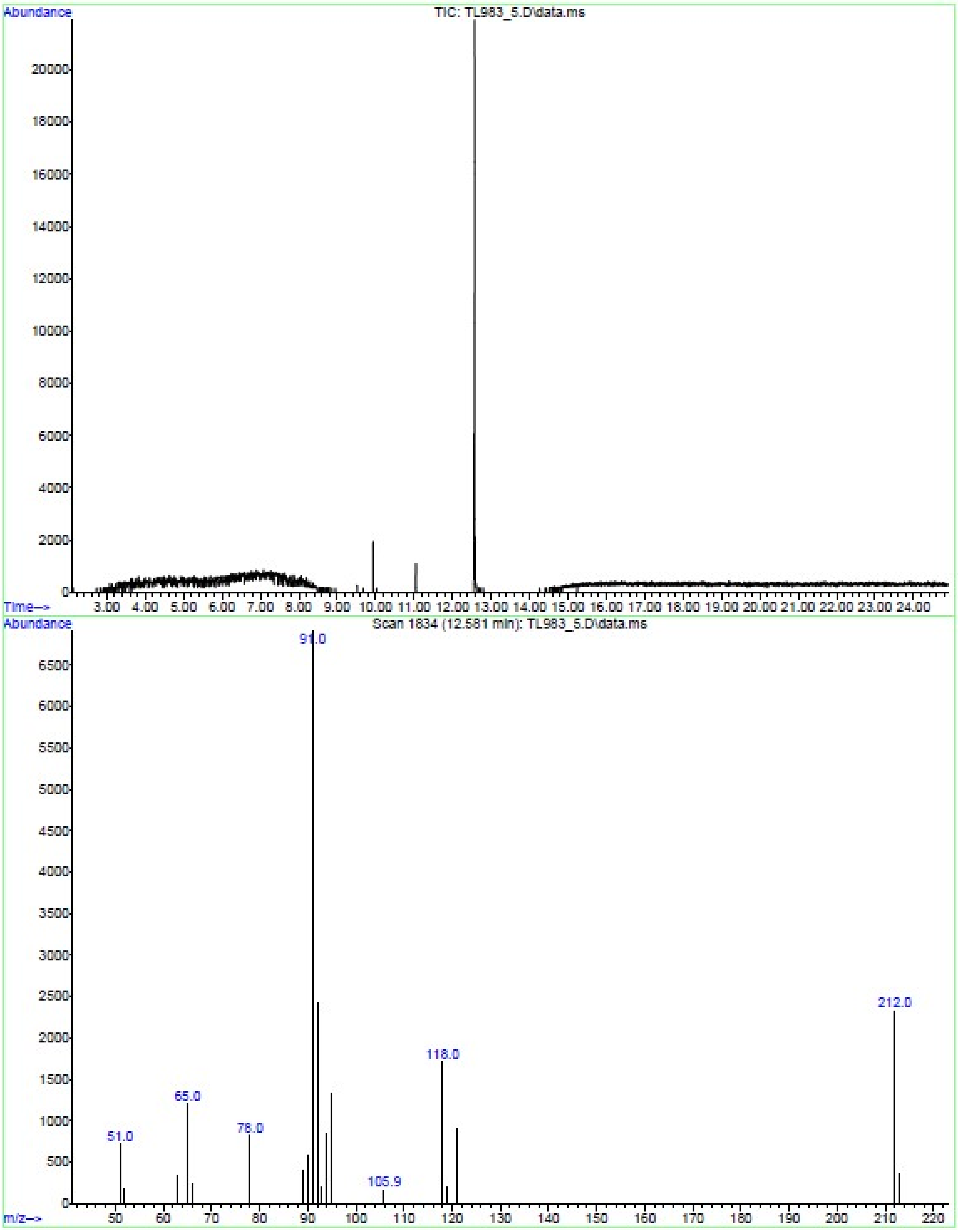


Figure S37 GC-MS of **19**

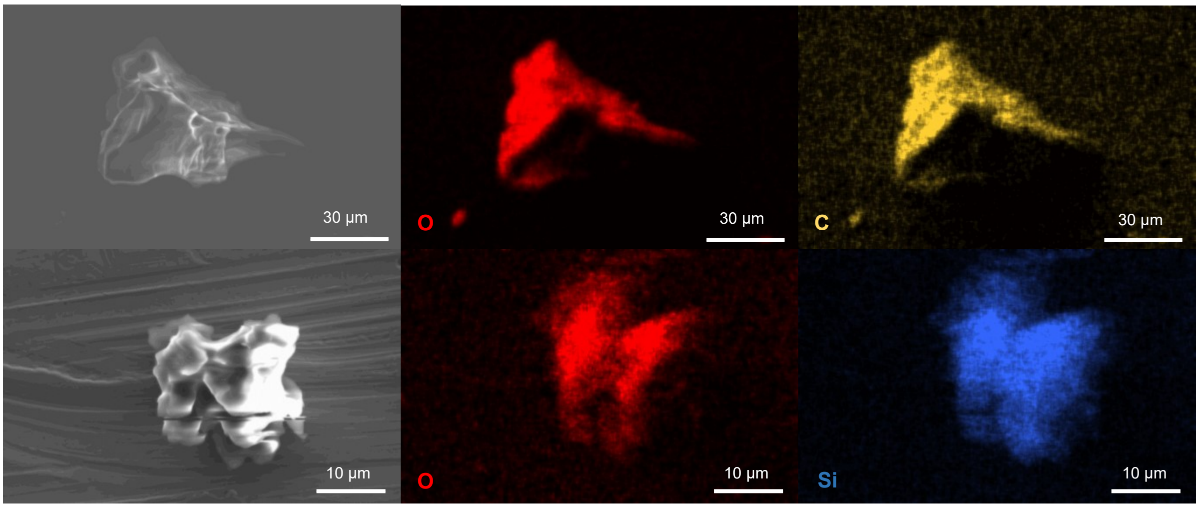


Figure S38 upper row: SEM/EDX mapping of the polysiloxane on a Si wafer; bottom row: SEM/EDX mapping of the polysiloxane on Cu tape.

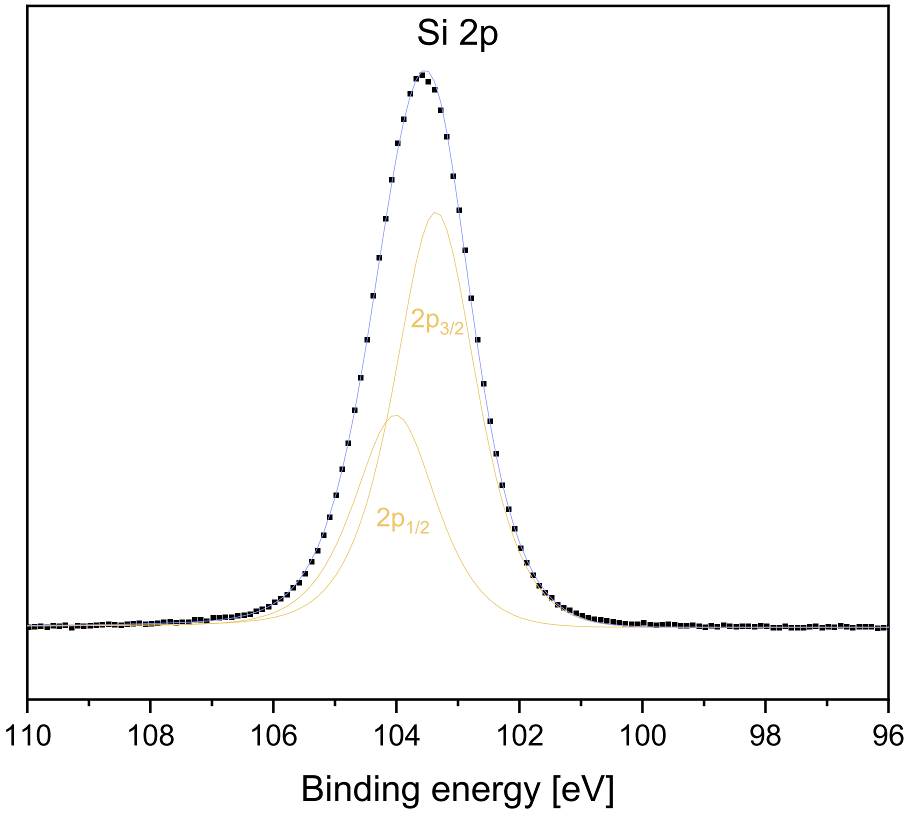


Figure S39 High-resolution XPS Si2p spectrum of the polysiloxane. Black squares show the experimental data, blue curve shows the envelope of the fit and the yellow curves show the fitted $2p_{3/2}$ and $2p_{1/2}$ areas. Fitting was carried out using Avantage Software of Thermo Scientific™.

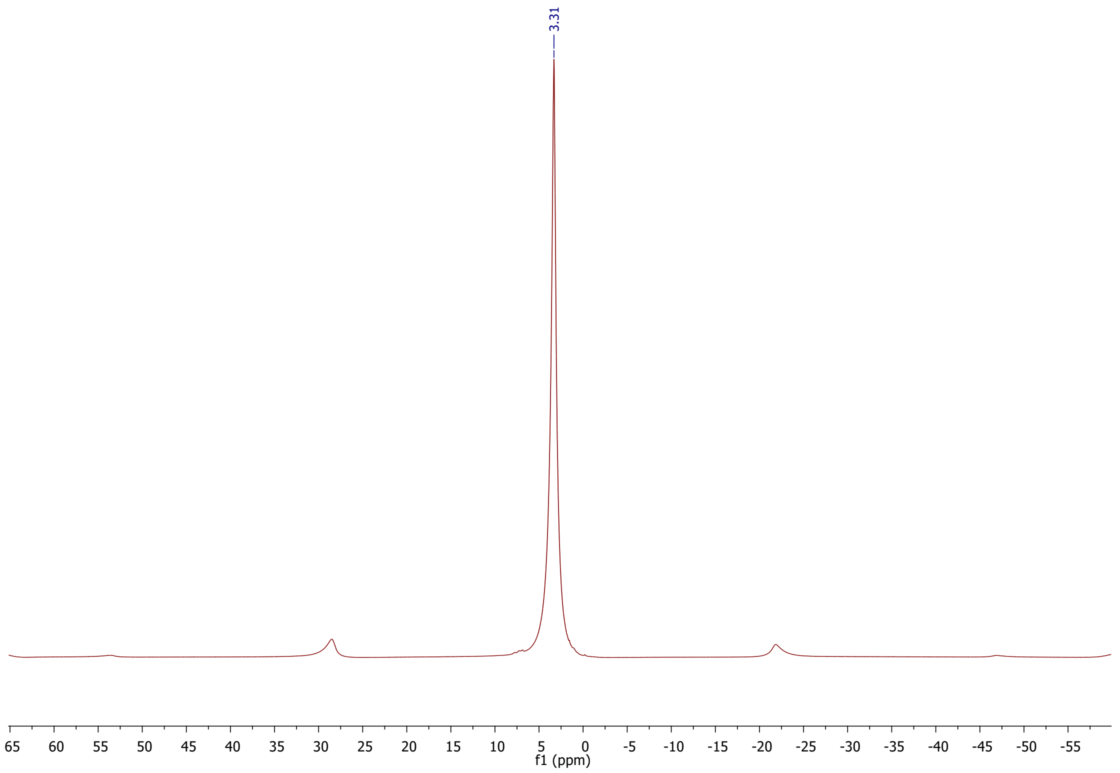


Figure S40 Solid state $^{1}H$ NMR spectrum of the polysiloxane.

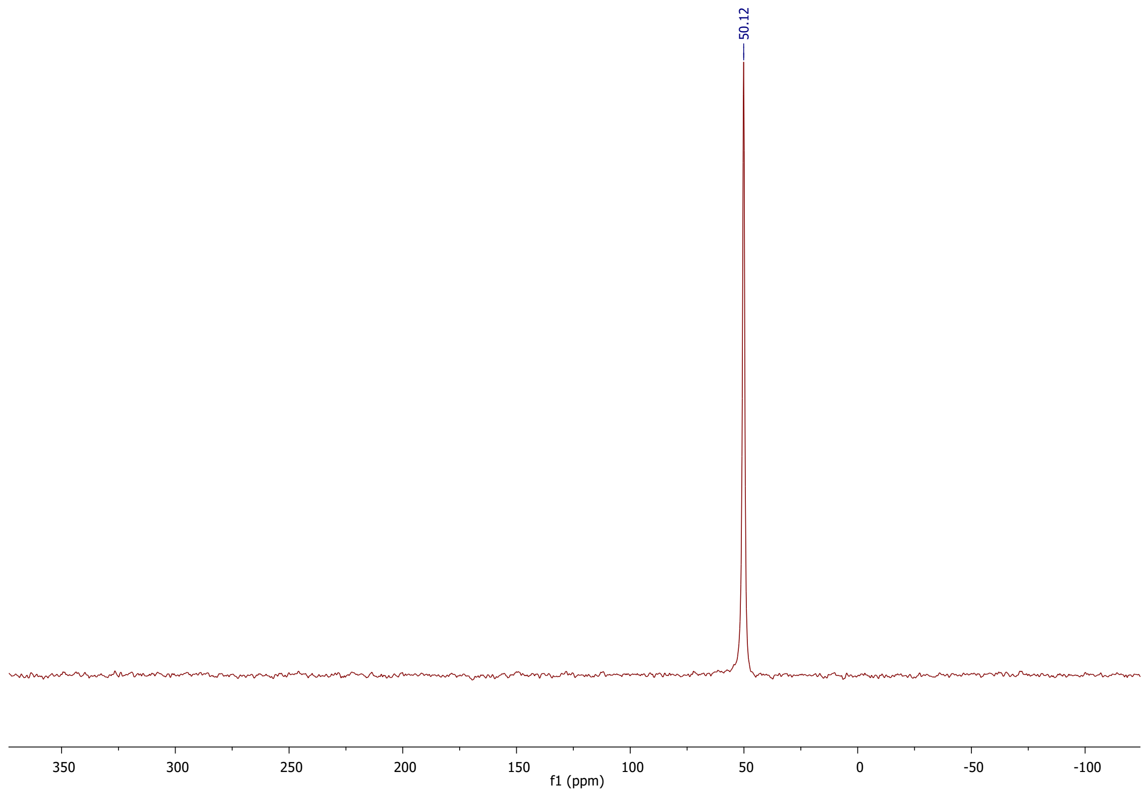


Figure S41 $^{13}C$ CPMAS NMR spectrum of the polysiloxane.

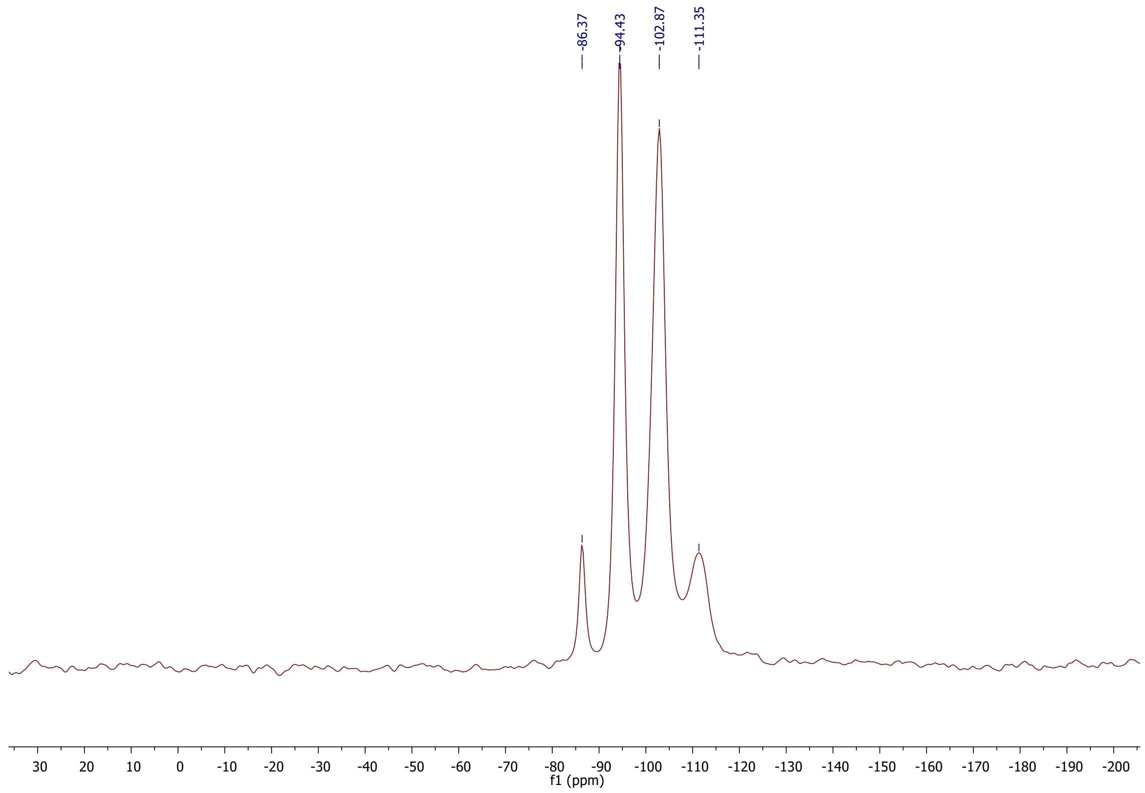


Figure S42 $^{29}$Si CPMAS NMR spectrum of the polysiloxane.

---

[1] L. U. Nordstrøm, H. Vogt, R. Madsen, *J. Am. Chem. Soc.* **2008**, *130*, 17672-17673

[2] Kita, Y.; Akai, S.; Ajimura, N.; Yoshigi, M.; Tsugoshi, T.; Yasuda, H.; Tamura, Y. J. Org. Chem.**1986**, 51, 4150–4158

[3] R. Paruszewski.; M. Strupinska., J. P .Stables.; M., Swiade.; S. Czuczwar, Z. Kleinrok, W. Turski *Chem. Pharm. Bull*. **2001**, 49, 629

[4] C.G. Jorgensen, B. Frolund, J. Kehler, A.A. Jensen, *ChemMedChem* **2011**, 6, 725 – 736

[5] D. N. Sawant, D. B. Bagal, S. Ogawa, K. Selvam and S. Saito, Org. Lett., **2018**, 20, 4397–4400

[6] L.-Y. Chen, M.-F. Wu, *Synthesis* **2019**, *51*, 1595-1602

[7] S. Jamalifard, J. Mokhtari, Z. Mirjafary, *RSC Adv.* **2019**, *9*, 22749-22754

[8] Tozawa, T.; Yamane, Y.; Mukaiyama, T. Chem. Lett. **2005**, 34, 1334–1335.

[9] Q. Shen, T. Ogata, J. Hartwig, *J. Am. Chem. Soc*. **2008**, 130, 20, 6586–6596

[10] T. Ohsima, T. Iwasaki, Y. Maegawa, A. Yoshiyama K. Mashima, *J. Am. Chem. Soc.* **2008**, 130, 10, 2944–2945

[11] J. hoerter, K. Otte, S. Gellman and S.Stahl, *J. Am. Chem. Soc*. 2006, 128, 15, 5177–5183

[12] S. Muthaiah, S. Chandra Ghosh, Joo-Eun Jee, C. Chen, J. Zhang, and S. Hyeok Hong *J. Org. Chem*. **2010**,75, 3002–3006

[13] S. Muthaiah, S. Chandra Ghosh, Joo-Eun Jee, C. Chen, J. Zhang, and S. Hyeok Hong *J. Org. Chem*. **2010**,75, 3002–3006
